# Dual activity of indolin-2-ones containing an arylidene motif: DNA and BSA interaction†

**DOI:** 10.1039/d3ra04997c

**Published:** 2023-09-25

**Authors:** Syed Nasir Abbas Bukhari, Tariq G. Alsahli, Hasan Ejaz, Naveed Ahmed, Waqas Ahmad, Mervat A. Elsherif, Nasser H. Alotaibi, Kashaf Junaid, Nenad Janković

**Affiliations:** a Department of Pharmaceutical Chemistry, College of Pharmacy, Jouf University Sakaka Al Jouf 72388 Saudi Arabia sbukhari@ju.edu.sa; b Department of Pharmacology, College of Pharmacy, Jouf University Sakaka Al Jouf 72388 Saudi Arabia TGAlsahli@ju.edu.sa; c Department of Clinical Laboratory Sciences, College of Applied Medical Sciences, Jouf University Sakaka Al Jouf 72388 Saudi Arabia hetariq@ju.edu.sa; d Department of Pharmaceutics, College of Pharmacy, Jouf University Sakaka Al Jouf 72388 Saudi Arabia nakahmad@ju.edu.sa; e Discipline of Pharmaceutical Chemistry, School of Pharmaceutical Sciences, Universiti Sains Malaysia Gelugor 11800 Penang Malaysia waqas@usm.my; f Chemistry Department, College of Science, Jouf University Sakaka Al Jouf 72388 Saudi Arabia maelsherif@ju.edu.sa; g Department of Clinical Pharmacy, College of Pharmacy, Jouf University Sakaka 72388 Saudi Arabia nhalotaibi@ju.edu.sa; h School of Biological and Behavioural Sciences, Queen Mary University of London London E1 4NS UK kashaf.junaid@qmul.ac.uk; i University of Kragujevac, Institute for Information of Technologies Kragujevac, Department of Science Jovana Cvijića bb 34000 Kragujevac Serbia nenad.jankovic@kg.ac.rs

## Abstract

Applying a multistep approach, novel indolin-2-ones (IND) that possess an arylidene motif have been synthesized. Eight compounds were chosen for different biological tests (antimicrobial and cytotoxicity). IND containing 2-thienyl (4h) fragment have been found to exhibit good antimicrobial activity against *B. cereus*. Molecules that have 3-aminophenyl (4d) or 2-pyridyl (4g) groups have shown the best antifungal activities against all tested fungi. These compounds have also been noticed as promising pharmaceuticals against MCF-7 cancer cell lines. Experimental outcomes from the investigation of the interaction of 4d with DNA implied its moderate binding to DNA (*K*_SV_ = 1.35 × 10^4^ and 3.05 × 10^4^ M^−1^ for EB and Hoechst binder, respectively). However, considerably stronger binding of 4d to BSA has been evidenced (*K*_a_ = 6.1 × 10^6^ M^−1^). In summary, IND that contains *m*-aminobenzylidene fragment (4d) exhibits a good dual biological activity making it a promising candidate for further investigation in the drug discovery sector.

## Abstract 2

The arylidene indolin-2-ones showed significant dual biological activity against MCF-T cancer cell lines and *Candida* species. The most promising molecule for further investigation contains an *m*-aminophenyl fragment.

## Graphical abstract

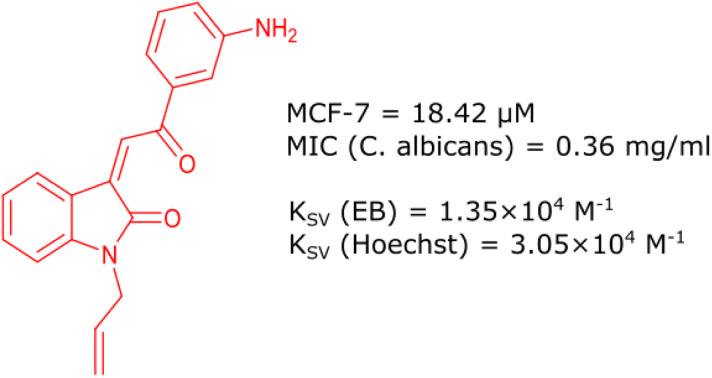

## Introduction

Indole and its derivatives have been used as synthetic intermediates or even equivalents in the production of diverse pharmaceuticals.^1^ Its chemical properties and reactivity make it very important in the chemical industry, where it is used as a starting material for the synthesis of many organic compounds, including dyes and agrochemicals.^2^ From a synthetic point of view and for drug discovery, 1*H*-indole-2,3-dione (isatin) is particularly important. In addition to its industrial applications, isatin is also found in nature as a component of many natural products, such as alkaloids and plant pigments. Along these lines, isatin is found in the genus *Isatis*^3^ in *Couroupita guianensis*^4^ and *Couroupita guianensis* Aubl.^5^ Substituted isatin has also been isolated from plant bodies such as *Melochia tomentosa*^6^ and the bacteria *Streptomyces albus*.^7^ In humans, isatin was found to be a metabolic derivative of adrenaline.^8^ It has also been studied for its potential biological activities. Indolin-2-one (IND) derivatives prepared from isatin have been shown to have various biological activities involving antimicrobial, antitumor, and anti-inflammatory properties, which make them very important for drug discovery and development.^1,2^

Some spiro-pyrazolo-3,3-oxindoles were synthesized by Abo-Salem and have shown promising anticancer activity against HCT-116 and MCF-7 cell lines, and the activities were like Doxorubicin.^9^ On the other hand, *N*-alkylated isatin connected with thiourea showed good potential against colon and multiple melanoma cancer cell lines. The investigated compound has shown ALDH inhibition potential and increased ROS activity.^10^ Interesting access was applied by Karthikeyan *et al.* Within this study, indol-2-one hybrids were interconnected to the chalcone pharmacologic motif. The achieved results were comparable to cis-platinum, with measured GI_50_ values in the range of 4.23–12.63 μM against MDA-MB468 and MCF-7 cell lines.^11^

**Fig. 1:**
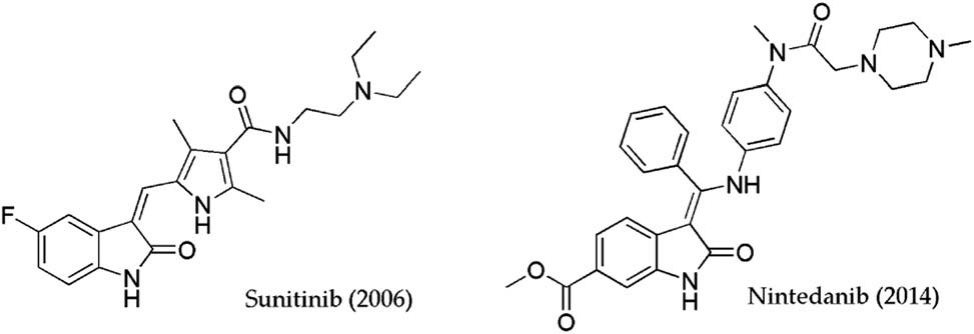
Indolin-2-one-based drugs approved by FDA.

Therefore, hydrazones of isatin have been shown to have promising anti-inflammatory activity at a dose level of 10 mg kg^−1^.^12^ Ibrahim *et al.* designed IND-based hydrazides with a diclofenac unit as part of the structure.^13^ In this study, diclofenac conjugates demonstrated a high percentage of inhibition of edema. Interesting antiviral activities against HIV1 and HIV2 were demonstrated by oxindole derivatives.^14–16^ 3-Arylidene-2-oxindole was denoted as GSK-3β inhibitors performed *in vitro* and *in vivo* experiments. Even antidiabetic activity authors suggested that arylidene-2-oxindole could be useful for the simultaneous treatment of two diseases cancer and diabetes.^17^ Based on biological evaluations that were obtained for chromone-isatin derivatives similar opinions were given by Wang and Rahim *et al.*^18,19^ Generally, multistep synthetic approaches open access to antimicrobial agents based on isatin or oxindole. In that sense, Biginelli's hybrids connected with isatin moiety showed antifungal activity against *A. niger* and *C. albicans* (MIC = 6–200 μg ml^−1^).^20^ 8-Methoxyl ciprofloxacin with isatin by linkage through the propylene chain was evaluated as an antibacterial agent. High activities comparable to vancomycin were achieved against Gram-positive bacteria with low MIC values (up to 0.25 μg ml^−1^). To date, the most useful IND-containing molecules that researchers have used as templates are the FDA-approved drugs Sunitinib and Nintedanib (Fig. 1).^21–24^ Sunitinib is the gold standard in the treatment of metastatic renal cell carcinoma, while Nintedanib is approved for use to treat idiopathic pulmonary fibrosis.^25–28^

During the last decade, significant progress has been made in the field of arylidene indolin-2-one (AIND) derivatives. The mentioned pharmacophores have been verified as antiplasmodial,^29^ tyrosine kinase inhibitors,^30^ radiotracers for Parkinson's disease detection,^31^ and antidepressants.^32^ Princiotto *et al.* investigated anticancer activity of selected 3-(hetero) AINDs against MCF-7 cell lines. Thiazole-containing 3-(hetero) AINDs have been the most promising activity (IC_50_ = 33 ± 2 μM).^33^ Very good selectivity followed with inhibitory potency against FGFR1 enzyme was declared for AINDs that contain morpholine or piperazine moiety.^34^ Furthermore, Senwar *et al.* pointed out the importance of the presence of an additional heterocyclic motif that positively affects the anticancer activity of AINDs. The presence of indolin-1-yl notably improves activity against prostate cancer cell lines (IC_50_ = 1.89 ± 0.6 μM) in comparison with morpholino, piperidin-1-yl, and pyrrolidine-1-yl fragments.^35^ Similar cytotoxicity study demonstrated that dimethylmorpholino and piperazin-1-yl tethered AINDs possessed good activity against breast cancer cell lines in the range 1.26–2.77 μM.^36^

The dual-activity principle in drug design usually refers to molecules that simultaneously have two different biological effects (*e.g.* anticancer and antimicrobial). The development of dual-active therapeutic entities has become a hot topic and emergency in the pharmaceutical industry. During the literature survey, we did not find that AIND has activity simultaneously against two different therapeutic targets. Considering, the main goal of this work was to synthesize a library of novel AIND derivatives and investigate their potential for dual biological activity. Our ongoing effort is to create novel potential bioactive compounds with benzylidene^37,38^ or indol-2-one units.^22,39^ The antimicrobial and antiproliferative effects of the IND-containing arylidene motif have been studied. In addition, for the most active compound under both biological tests, interactions with biomacromolecules (DNA and BSA) were investigated.

## Results and discussion

### Synthesis and characterization

**Scheme 1:**
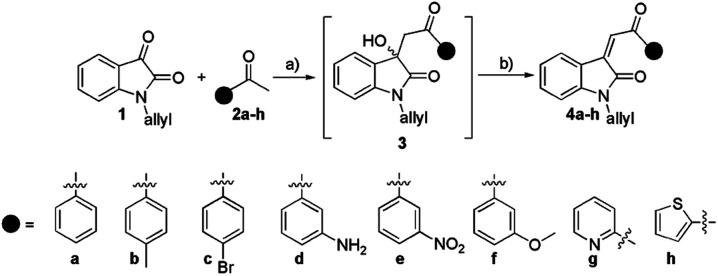
The synthesis of arylidene isatin-2-ones 4a–h: (a) Et_2_NH, abs. ethanol; (b) glacial acetic acid, *cc.* HCl.

Our initial experiments began with the synthesis of the precursor *N*-allyl isatin (1). Molecule 1 was prepared using an earlier described method with a small modification.^40^ In the next synthetic step, 1 was subjected to a reaction with aromatic ketones 2a–h under basic catalytic conditions (Et_2_NH). During this step, the significant issue was reaching the Knoevenagel adduct in a single step. None of the attempts gave the final product 4 even after 24 hours. The product of aldol addition 3 was isolated and structurally confirmed in only one case. Namely, in the reaction of acetophenone (2a) and 1, the product of aldol addition (3) precipitates from the solution. Applying other aromatic ketones 2b–h we failed to isolate the target adduct 3. Upon reaction between 1 and 2 solvent was evaporated, and then the crude mixture was stirred with glacial acetic acid and *cc.* HCl (10 : 1, v/v). After the work-up, 4a–h products were characterized using IR, NMR, elemental analysis, and MS. The yields of products 4a–h were in the range of 27–81%. The best yield was obtained for 4e (81%). Compounds 4c–4g are described for the first time (Scheme 1).

Generally, IR spectra typically exhibit strong absorption bands of unsaturated ketones in the range of 1700–1600 cm^−1^. In general, around 1710 cm^−1^ is the band for the stretching of the amide carbonyl (N–C=O) group as part of a five-membered lactam. The middle bands located at higher frequencies 3100–3000 cm^−1^ are attributed to the –C=C–H vibration. Especially, in the IR spectrum of 4d characteristic absorption for primary amines in the range 2800–2550 cm^−1^ were displayed.^41^ Free primary amine vibration frequencies were found at 2803 and 2572 cm^−1^. In proton NMR spectra of 4a–h typically sharp and intensive singlet was located at ≈7.8 ppm that originated from the resonance of exocyclic double bond proton =CH. Carbon NMR of 4a–h showed characteristic resonance of keto carbonyl C=O in the range 183–193 ppm. Amide carbonyl and quaternary carbon of the exocyclic double bond (C_q_=CH) showed resonance at ≈167 ppm and ≈145 ppm, respectively. Additional characterization for all samples was done using ESI-MS techniques (Fig. S19–S26†). Characteristic molecular ions of 4a–h molecules were found. Also, in some specific cases, cluster ions were also found. For instance, molecule 4a had molecular ion [M + H]^+^ = 290 *m*/*z*, and cluster ion [M + M + H]^+^ = 579 *m*/*z*. Therefore, upon ionization in all samples fragment ions at 214 *m*/*z* were obtained. Most likely, mentioned arise because of the fragmentation of the O=C–aromatic ketone bond.^42^

### Biological activity

AIND derivatives 4a–h were exposed to different biological tests (antimicrobial and antifungal). For these experiments, we treated different Gram-positive (*Bacillus subtilis*, *Bacillus cereus*, *Staphylococcus aureus*, and *Enterococcus faecalis*) and Gram-negative bacteria (*Escherichia coli*, *Proteus mirabilis*, and *Klebsiella pneumoniae*). The antifungal potential was checked against six fungi *Cladosporium cladosporioides*, *Penicillium italicum*, *Aspergillus niger*, *Candida albicans*, *Candida krusei* and *Candida parapsilosis*. Among them, three species of *Candida* belong to the group of the most common fungi responsible for superficial and systemic infections.^43,44^ The two most active AINDs were subjected to cytotoxicity tests against cancer (A549, LS174, MCF-7, and PaCa-2) and normal cell lines (MRC-5).

The results of antimicrobial and cytotoxicity testing are shown in Tables 1–3 and present the average of selected activities of indolin-2-ones 4a–h defined in triplicate.

**Table 1:** Antibacterial activity of 4a–h. MIC values is given in mg ml^−1^

	*E. coli*	*P. mirabilis*	*K. pneumoniae*	*B. subtilis*	*S. aureus*	*E. faecalis*	*B. cereus*
4a	12.5	12.5	25	25	12.5	25	25
4b	12.5	25	25	12.5	25	25	25
4c	6.25	6.25	25	12.5	12.5	25	12.5
4d	12.5	6.25	12.5	12.5	25	6.25	25
4e	12.5	12.5	12.5	6.25	12.5	25	25
4f	25	12.5	3.1	6.25	25	25	25
4g	25	25	3.1	3.1	12.5	25	25
4h	25	12.5	3.1	3.1	6.25	12.5	12.5
Streptomycin	0.062	0.062	0.031	0.016	0.031	0.031	0.016

**Table 2:** Antifungal activity of 4a–h. MIC values is given in mg ml^−1^

	*C. cladosporioides*	*C. albicans*	*C. krusei*	*C. parapsilosis*	*P. italicum*	*A. niger*
4a	6.25	6.25	12.5	25	12.5	6.25
4b	12.5	6.25	12.5	25	6.25	6.25
4c	12.5	6.25	12.5	6.25	12.5	12.5
4d	0.36	0.36	6.25	6.25	0.36	0.36
4e	12.5	12.5	12.5	12.5	12.5	12.5
4f	12.5	12.5	3.1	6.25	12.5	12.5
4g	6.25	3.1	3.1	3.1	6.25	3.1
4h	12.5	25	25	25	6.25	3.1
Fluconazole	0.39	0.78	0.78	0.39	0.78	0.39

**Table 3:** Antiproliferative effect of most active AINDs (IC_50_ in μM)

	A549	LS174	MCF-7	PaCa-2	MRC-5
4d	87.41 ± 1.59	121.38 ± 3.17	18.42 ± 0.45	140 ± 1.74	107.29 ± 1.51
4g	143.75 ± 2.40	101.85 ± 1.06	25.19 ± 0.62	128 ± 2.35	104.12 ± 2.86
cisPt	11.59 ± 1.64	4.83 ± 0.35	18.05 ± 1.28	11.34 ± 1.36	9.35 ± 1.29

Results of antimicrobial and antiproliferative screening of indolin-2-one derivatives 4a–h are presented in Tables 1–3. According to the results of antimicrobial testing, all tested compounds were moderately active against both Gram-positive and Gram-negative bacteria (Table 1). For example, 4f, 4g, and 4h had noticeably greater efficacy against *K. pneumoniae* Gram-negative strains (MIC = 3.125 mg ml^−1^). Generally, experimental deliverables from the antifungal study implied that indolin-2-ones 4a–h possessed better antifungal than antibacterial potential. MIC values were in the range of 0.36–25 mg ml^−1^. 2-Pyridyl fragment (4g) has been crucial for very good anticandida activities. Indolin-2-one with pyridyl moiety showed the highest activity against *C. albicans*, *C. krusei*, and *C. parapsilosis* (Table 2). For all three *Candida* species have been achieved MIC values 3.1 mg ml^−1^. Compound 4d which contains 3-aminophenyl function has shown the highest activities against *Cladosporium cladosporioides*, *C. albicans*, *P. italicum*, and *A. niger* fungi strains. Considering results from antimicrobial screening, compounds 4d and 4g were used as the most active and were exposed to antiproliferative activity (Table 3).

The antiproliferative potential of selected molecules was investigated against both normal (MRC-5) and malignant cell lines (A549, LS174, MCF-7, and PaCa-2). Table 3 displays the experimental findings that are given as an IC_50_ value. While 4d and 4g have good activities against MCF-7, on other cancer cell lines they have a mild antiproliferative impact. 4d reached the lowest IC_50_ and consequently the highest antiproliferative action (18.42 ± 0.45 μM). Investigation on normal MRC-5 cell lines suggests no significant toxicity of both compounds 4d and 4g. A good selectivity index for 4d and 4g was achieved.

The antiproliferative evaluation of related AINDs revealed diverse actions that were strongly dependent on the studied compounds structures. The 6-methoxy group at the indolin-2-one ring that relates to the *m*-methoxybenzylidene motif showed very strong activity against MCF-7 cell lines (IC_50_ = 1.2 ± 0.4 μM), but without selectivity. Indolin-2-one with *m*-methoxy- or *m*-methylbenzylidene function has shown limited activity against Hepa1c1c7 cell lines (IC_50_ > 25 μM) due to the absence of an alkyl group at the nitrogen position.^45^ This is also in accordance with investigation of the Yang *et al.*, where authors proposed that anticancer actions are strongly dependent by the presence of *N*-substituent.^46^ The addition of a *N*-aryl fragment to the structure of indolin-2-ones affects their anticancer activity. The activities against K562 cell lines were significantly improved in the presence of *N*-phenyl and -methoxy functions (IC_50_ = 31.68 μM). Despite this, the introduction of *m*-aminobenzylidene or 2-pyridyl and an allyl fragment in the indolin-2-one structure had a significant influence on biological activities (Tables 1–3). Compound 4d contains an *m*-aminobenzylidene motif and has demonstrated antibacterial and anticancer activity, particularly against MCF-7 cell lines (IC_50_ = 18.42 ± 0.45 μM). Furthermore, molecule 4g includes a pyridyl motif and demonstrated very good anticancer activity against the same cancer cell lines. In comparison to the positive control, cis-platinum, both compounds have higher and positive selectivity (Table 3). From structural point of view, *m*-aminobenzylidene (4d) or 2-pyridyl (4g) motif have the potential to make hydrogen bonds. It could be the reason for the significant difference between biological activities of 4d and 4g in comparison with other synthesized indolin-2-ones.

### Interactions with biomacromolecules (DNA and BSA)

**Fig. 2:**
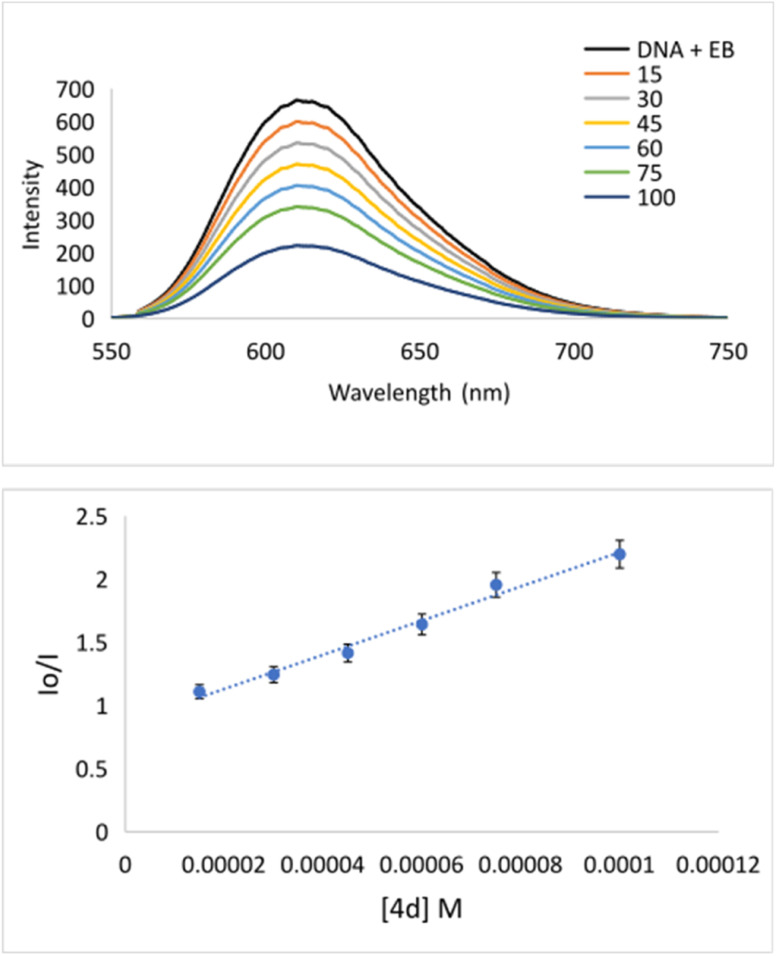
(Top) Emission titration of DNA–EB with 4d. The black line denotes solution: buffer + DNA–EB. [EB] = 100 μM, [DNA] = 100 μM; [4d] = 0–100 μM; pH = 7.4; *λ*_ex_ = 520 nm, *λ*_em_ = 611 nm; (Bottom) plot of *I*_0_/*I versus* [4d].

The interaction of potential pharmaceuticals with DNA can occur in several ways that generally depend on the type of structural motif. The most common interaction mode is intercalation. Under this process, molecules, or even ions are inserted between the base pairs of DNA. Many chemotherapeutics acts by intercalating into the DNA of cancer cells, disrupting the normal cell division process, and ultimately causing the death of cancer cell.^47,48^ Because of the importance of mode of action, the interaction of 4d with DNA was explored using fluorescence spectroscopy (Fig. 2). Apart from this, bovine serum albumin is a commonly used protein in biological research, and it is often used as a model carrier protein to study drug–protein interactions. The binding potential of pharmaceuticals to BSA can provide insights into how the molecule interacts with carrier proteins in the body. To bind a molecule to BSA, it is typically necessary to mix the potential drug with a solution of BSA and incubate the mixture for 12 h. During this time, the investigated compound will bind to the BSA forming drug–BSA complex species.

The binding could be quantified using fluorescence spectroscopy.^49^ Considering the importance of understanding the binding of a drug to BSA, we have investigated the potential for the formation of a 4d–BSA complex employing the emission titration method (Fig. 4). All measurements were done in triplicate. During fluorescence titration of preformed DNA–EB species with quencher 4d emission spectra, they were recorded (Fig. 2). The fluorescence emissions of 4d were captured between 550 and 750 nm (Fig. 2). The intensity of the fluorescence emission line of DNA–EB significantly decreased during the addition of increasing concentrations of 4d to DNA–EB. This observed quenching implies that the molecules 4d and EB are competitive for DNA binding.

**Fig. 3:**
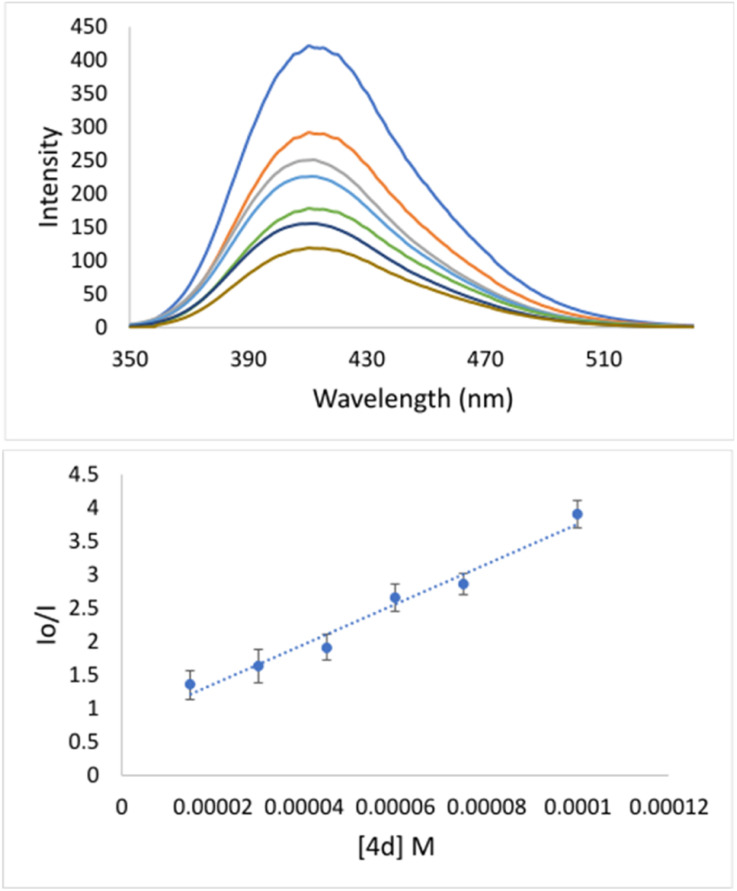
(Top) Emission titration of DNA–Hoechst with 4d. The blue line denotes solution: buffer + DNA–Hoechst. [Hoechst] = 100 μM, [DNA] = 100 μM; [4d] = 0–100 μM; pH = 7.4; *λ*_ex_ = 340 nm, *λ*_em_ = 410 nm; (Bottom) plot of *I*_0_/*I versus* [4d].

Specifically, the Stern–Volmer constant (*K*_SV_) indicates moderate binding to DNA. Even though 4d is quite a rigid and planar molecule, its surprisingly low *K*_SV_ value indicates no intercalation action between base pairs on the major groove side. Therefore, low *K*_SV_ combined with high *k*_q_ indicates minor groove binding. The quenching constant (1.35 × 10^12^) has been higher than the maximum scattering collision quenching constant (2.0 × 10^10^ l mol^−1^) implying static quenching.^50^ Measured *K*_SV_ of 4d implies its similar mechanism of binding to DNA as it was declared for well-known indole-based nonsteroidal anti-inflammatory drug Indomethacin.^51^ Going deeper inside of groove binding, minor groove binder Hoechst 33258 (Hoechst) has been applied.^52^ As can be seen from Fig. 3, after adding of 4d into DNA–Hoechst solution (blue line) emission intensity line decreased rapidly. Stern–Volmer constant is 3.05 × 10^4^ M^−1^ (Table 4). The constant is higher in comparison with EB quencher (*K*_SV_ = 1.35 × 10^4^ M^−1^) indicating that Hoechst was replaced with 4d much easier then EB. The mentioned fact indicates minor groove binding of 4d.^51^

**Table 4:** Binding parameters of 4d

	*K*_SV_ [M^−1^]		*k*_q_ [M^−1^ s^−1^]	*K*_a_ [M^−1^]	*n*
	EB	Hoechst			
4d	1.35 × 10^4^	3.05 × 10^4^	1.35 × 10^12^	6.1 × 10^6^	1.1

**Fig. 4:**
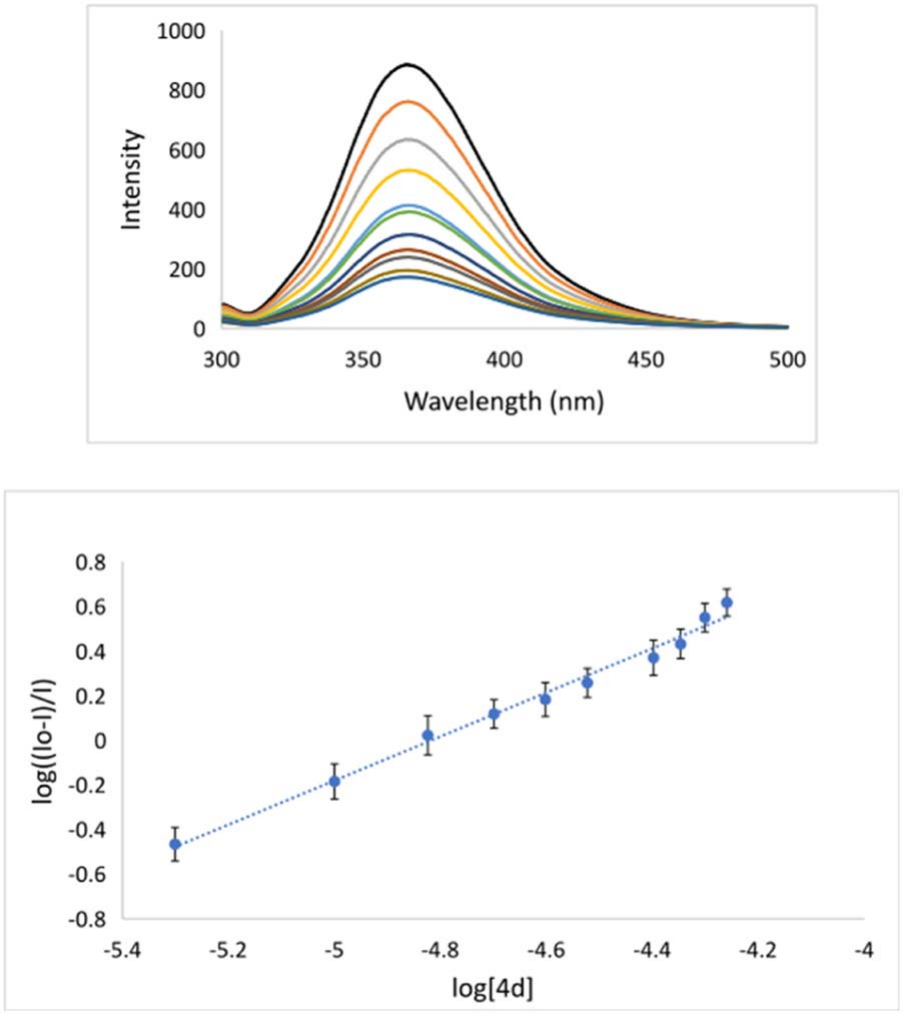
(Top) Emission titration of BSA with 4d. The black lines denote solution of [BSA] = 5 μM, [4d] = 0.0, 5.0, 10.0, 15, 20, 25, 30, 35, 40, 45 and 50; pH = 7.4; *λ*_ex_ = 287 nm; (Bottom) plot of log(*I*_0_ − *I*/*I*) *versus* log[4d].

The binding constant (*K*_a_ = 6.1 × 10^6^ M^−1^) implies that 4d binds sufficiently strongly to BSA (Table 4 and Fig. 4). This experimental fact indicates that 4d can form the 4d–BSA cluster and should be successfully carried out by BSA to selected pharmacological targets.

## Experimental

All necessary chemicals were supplied by Sigma Aldrich. The IR spectra were recorded by a PerkinElmer Spectrum One FT-IR spectrometer (USA) on a KBr pellet. Melting points (mp) were measured at Bibby Scientific Limited SMP10 (UK). Elemental analyses were performed using a Vario Elemental Analyser Leco CHNS-932 (UK). Fluorescence measurements were conducted at the RF-6000 PC spectrofluorometer (Shimadzu, Japan). NMR spectra were recorded on a Varian Gemini 200 MHz NMR spectrometer (USA) in DMSO-*d*_6_ as solvent. Signals are described as s = singlet, d = doublet, and m = multiplet. Proton and carbon NMR (Fig. S1–S18†), MS (Fig. S19–S26†), and IR spectra (Fig. S27–S34†) are given in ESI.† Bacteria, fungi, and cancer cell lines (A549, LS174, PaCa-2, and MCF-7) were from the American Type Culture Collection (ATCC). Antimicrobial activities were investigated by performing the broth dilution method.^53^ Concentrations of the investigated compounds were in the range of 25–0.0195 mg ml^−1^. The minimal inhibitory concentration (MIC) was established using 96-well microtiter plates.^54^ Cytotoxicity tests and MTT assays of selected compounds were done following the described protocol.^22^ All procedures were performed in triplicate. Cis-platinum, fluconazole, and streptomycin were used as references.

### Synthesis of indolin-2-one derivatives (4)

Allyl isatin (5 mmol) and an excess of various ketones (7 mmol) were dissolved in 30 ml of absolute ethanol. Diethylamine (0.5 ml) was used as a catalyst. Reactions were refluxed for 12–24 hours. Upon selected reaction time, reaction mixtures were evaporated. In the dark red reaction crude, 10 ml of freshly distilled glacial acetic acid and 1 ml of HCl (36%) were added and refluxed over 6 hours. The hot mixture was poured into the crushed ice/^i^PrOH mixture. The precipitated product was filtrated and washed with 50% ^i^PrOH. 4a–h has been isolated in good yields. Short column was used for purification of samples 4a–f (eluent DCM). Experimental data for 4a–h are given below.

### Experimental data

#### 1-Allylindoline-2,3-dione 1

Red solid; yield: 96%; mp = 95 °C; ^1^H NMR (200 MHz, DMSO-d_6_) *δ* 7.68–7.53 (m, 2H, CH_Ar_), 7.16–7.03 (m, 2H, CH_Ar_), 5.96–5.77 (m, 1H, =CH), 5.39–5.16 (m, 2H, =CH_2_), 4.31 (dt, *J* = 4.9, 1.7 Hz, 2H, N–CH_2_) ppm; ^13^C NMR (50 MHz, DMSO-d_6_) *δ* 183.2, 157.9, 150.5, 138.1, 131.4, 124.5, 123.3, 117.5, 111.2, 41.9 ppm.

#### 1-Allyl-3-(2-oxo-2-phenylethylidene)indolin-2-one 4a

Orange solid; yield: 54% (1.06 g); mp = 85 °C; IR *ν* 3434, 1706, 1658, 1619, 1600, 1464, 1346, 1225 cm^−1^; ^1^H NMR (200 MHz, DMSO-d_6_) *δ* 8.11–8.03 (m, 3H, CH_Ar_), 7.81 (s, 1H, =CH), 7.77–7.68 (m, 1H, CH_Ar_), 7.63–7.55 (m, 2H, CH_Ar_), 7.44–7.35 (m, 1H, CH_Ar_), 7.04–6.97 (m, 2H, CH_Ar_), 5.97–5.78 (m, 1H, =CH_allyl_), 5.24–5.14 (m, 2H, =CH_2_), 4.39–4.37 (m, 2H, N–CH_2_) ppm; ^13^C NMR (50 MHz, DMSO-d_6_) *δ* 191.3, 166.5, 144.9, 136.9, 135.0, 134.2, 132.7, 131.8, 129.2, 128.7 (2C), 127.1 (2C), 126.4, 122.4, 119.4, 117.1, 109.8, 41.9 ppm; ESI-MS (*m*/*z*): [M + H]^+^ = 290; elemental analysis (%): cacld for C_19_H_15_NO_2_: C 78.87; H 5.23; N 4.84; found: C 78.64; H 5.12; N 4.69.

#### 1-Allyl-3-(2-oxo-2-*p*-tolylethylidene)indolin-2-one 4b

Orange solid; yield: 49% (1.13 g); mp = 90–92 °C; IR *ν* 3434, 1714, 1657, 1615, 1605, 1465, 1360, 1348 cm^−1^; ^1^H NMR (200 MHz, DMSO-d_6_) *δ* 8.01–7.95 (m, 3H, CH_Ar_), 7.79 (s, 1H, =CH), 7.41–7.32 (m, 3H, CH_Ar_), 7.03–6.96 (m, 2H, CH_Ar_), 6.01–5.78 (s, 1H, =CH_allyl_), 5.23–5.14 (m, 2H, CH_2_), 4.37 (d, *J* = 5.0 Hz, 2H, N–CH_2_), 2.39 (s, 3H, CH_3_) ppm; ^13^C NMR (50 MHz, DMSO-d_6_) *δ* 190.8, 166.6, 144.9, 144.8, 134.7, 134.5, 132.6, 131.8, 129.8 (2C), 128.8 (2C), 127.4, 126.3, 122.3, 119.4, 117.1, 109.7, 41.9, 21.5 ppm; ESI-MS (*m*/*z*): [M + H]^+^ = 304; cacld for C_20_H_17_NO_2_: C 79.19; H 5.65; N 4.62; found: C 79.02; H 5.48; N 4.50.

#### 1-Allyl-3-(2-(4-bromophenyl)-2-oxoethylidene)indolin-2-one 4c

Red solid; yield: 80% (2.21 g); mp = 98 °C; IR *ν* 3432, 1716, 1665, 1616, 1596, 1466 cm^−1^; ^1^H NMR (200 MHz, DMSO-d_6_) *δ* 8.07–7.97 (m, 3H, CH_Ar_), 7.97–7.70 (m, 3H, =CH + CH_Ar_), 7.45–7.35 (m, 1H, CH_Ar_), 7.06–6.98 (m, 2H, CH_Ar_), 6.02–5.78 (s, 1H, =CH_allyl_), 5.23–5.14 (m, 2H, CH_2_), 4.37 (d, *J* = 5.0 Hz, 2H, N–CH_2_) ppm; ^13^C NMR (50 MHz, DMSO-d_6_) *δ* 190.3, 166.5, 145.1, 136.0, 135.5, 132.9 (2C), 132.2 (2C), 131.8, 130.6, 128.4, 126.6, 126.4, 122.4, 119.4, 117.2, 109.8, 41.9 ppm; ESI-MS (*m*/*z*): [M]^+^ = 368; cacld for C_19_H_14_BrNO_2_: C 61.97; H 3.83; N 3.80; found: C 61.70; H 3.64; N 3.72.

#### 1-Allyl-3-(2-(3-aminophenyl)-2-oxoethylidene)indolin-2-one 4d

Dark red crystals; yield: 43% (1.15 g); mp = 168 °C; IR *ν* 3435, 2803, 2752, 2572, 1710, 1657, 1614, 1599, 1466, 1256 cm^−1^; ^1^H NMR (200 MHz, DMSO-d_6_) *δ* 8.07 (d, *J* = 7.4 Hz, 1H, CH_Ar_), 8.02–7.96 (m, 2H, CH_Ar_), 7.77 (s, 1H, =CH), 7.66–7.60 (m, 2H, CH_Ar_), 7.45–7.37 (m, 1H, CH_Ar_), 7.06–6.98 (m, 2H, CH_Ar_), 5.96–5.78 (m, 1H, =CH_allyl_), 5.22–5.13 (m, 2H, =CH_2_), 4.38 (d, *J* = 5.0 Hz, 2H, N–CH_2_) ppm; ^13^C NMR (50 MHz, DMSO-d_6_) *δ* 190.4, 166.6, 145.1, 138.1, 136.0, 135.6, 133.0, 131.8, 130.6, 127.3, 126.6, 126.5, 126.2, 122.5, 121.4, 119.4, 117.2, 109.9, 41.9 ppm; ESI-MS (*m*/*z*): [M + H]^+^ = 305; cacld for C_19_H_16_N_2_O_2_: C 74.98; H 5.30; N 9.20; found: C 74.82; H 5.15; N 9.28.

#### 1-Allyl-3-(2-(3-nitrophenyl)-2-oxoethylidene)indolin-2-one 4e

Brown solid; yield: 81% (2.10 g); mp = 130 °C; IR *ν* 3428, 1711, 1657, 1609, 1530, 1467, 1346 cm^−1^; ^1^H NMR (200 MHz, DMSO-d_6_) *δ* 8.73–8.71 (m, 1H, CH_Ar_), 8.51–8.48 (m, 2H, CH_Ar_), 8.16 (d, *J* = 7.4 Hz, 1H, CH_Ar_), 7.92–7.82 (m, 2H, =CH + CH_Ar_), 7.48–7.39 (m, 1H, CH_Ar_), 7.08–6.99 (m, 2H, CH_Ar_), 6.04–5.79 (m, 1H, =CH_allyl_), 5.24–5.15 (m, 2H, =CH_2_), 4.40–4.38 (m, 2H, N–CH_2_) ppm; ^13^C NMR (50 MHz, DMSO-d_6_) *δ* 189.2, 166.5, 148.2, 145.4, 138.2, 136.5, 134.8, 133.4, 131.7, 131.0, 128.1, 127.0, 125.4, 122.9, 122.5, 119.3, 117.2, 109.8, 41.9 ppm; ESI-MS (*m*/*z*): [M + H]^+^ = 335; cacld for C_19_H_14_N_2_O_4_: C 68.26; H 4.22; N 8.38; found: C 68.10; H 4.20; N 8.25.

#### 1-Allyl-3-(2-(3-methoxyphenyl)-2-oxoethylidene)indolin-2-one 4f

Orange amorphous solid; yield: 64% (1.31 g); mp = 80 °C; IR *ν* 3434, 1715, 1657, 1620, 1591, 1462, 1354, 1262 cm^−1^; ^1^H NMR (200 MHz, DMSO-d_6_) *δ* 8.03 (d, *J* = 7.3 Hz, 1H, CH_Ar_), 7.78 (s, 1H, =CH), 7.69–7.63 (m, 1H, CH_Ar_), 7.54–7.26 (m, 4H, CH_Ar_), 7.05–6.97 (m, 2H, CH_Ar_), 6.02–5.76 (m, 1H, =CH_allyl_), 5.23–5.14 (m, 2H, =CH_2_), 4.37 (d, *J* = 5.0 Hz, 2H, N–CH_2_), 3.84 (s, 3H, OCH_3_) ppm; ^13^C NMR (50 MHz, DMSO-d_6_) *δ* 191.0, 166.6, 159.7, 145.0, 138.3, 135.1, 132.8, 131.8, 130.4, 127.1, 126.4, 122.4, 121.5, 120.4, 119.4, 117.2, 112.6, 109.8, 55.6, 41.9 ppm; ESI-MS (*m*/*z*): [M + H]^+^ = 320; cacld for C_20_H_17_NO_3_: C 75.22; H 5.37; N 4.39; found: C 75.12; H 5.26; N 4.21.

#### 1-Allyl-3-(2-oxo-2-(pyridin-2-yl)ethylidene)indolin-2-one 4g

Dark red crystals; yield: 27% (0.6 g); mp = 118–120 °C; IR *ν* 3434, 1704, 1668, 1619, 1597, 1466, 1363, 1226 cm^−1^; ^1^H NMR (200 MHz, DMSO-d_6_) *δ* 8.82–8.87 (m, 1H, CH_Ar_), 8.55–8.51 (m, 1H, CH_Ar_), 8.45 (s, 1H, =CH), 8.14–8.07 (m, 2H, CH_Ar_), 7.74–7.72 (m, 1H, CH_Ar_), 7.43–7.39 (m, 1H, CH_Ar_), 7.06–6.96 (m, 2H, CH_Ar_), 5.96–5.77 (m, 1H, =CH_allyl_), 5.22–5.13 (m, 2H, =CH_2_), 4.38–4.35 (m, 2H, N–CH_2_) ppm; ^13^C NMR (50 MHz, DMSO-d_6_) *δ* 190.0, 166.9, 153.4, 149.3, 145.5, 138.0, 136.5, 133.4, 131.8, 128.2, 127.6, 124.9, 122.6, 122.5, 119.7, 117.1, 109.7, 41.9 ppm; ESI-MS (*m*/*z*): [M + H]^+^ = 291; cacld for C_18_H_14_N_2_O_2_: C 74.47; H 4.86; N 9.65; found: C 74.28; H 4.71; N 9.49.

#### 1-Allyl-3-(2-oxo-2-(thiophen-2-yl)ethylidene)indolin-2-one 4h

Red solid; yield: 27% (0.64 g); mp = 119–121 °C; IR *ν* 3428, 1709, 1643, 1613, 1464, 1413, 1352 cm^−1^; ^1^H NMR (200 MHz, DMSO-d_6_) *δ* 8.33 (d, *J* = 7.8 Hz, 1H, CH_Ar_), 8.18–8.13 (m, 2H, CH_Ar_), 7.74 (s, 1H, =CH), 7.43 (td, *J* = 7.8, 1.3 Hz, 1H, CH_Ar_), 7.32 (dd, *J* = 4.9, 3.9 Hz, 1H, CH_Ar_), 7.09–6.98 (m, 2H, CH_Ar_), 5.97–5.78 (m, 1H, =CH_allyl_), 5.23–5.13 (m, 2H, =CH_2_), 4.40–4.36 (m, 2H, N–CH_2_) ppm; ^13^C NMR (50 MHz, DMSO-d_6_) *δ* 182.6, 166.6, 145.2, 144.9, 137.0, 135.8, 134.7, 133.1, 131.8, 129.4, 127.4, 125.5, 122.4, 119.5, 117.1, 109.7, 41.9 ppm; ESI-MS (*m*/*z*): [M + H]^+^ = 296; cacld for C_17_H_14_NO_2_S: C 68.90; H 4.76; N 4.73; found: C 68.82; H 4.57; N 4.63.

### MTT assay

The inhibitory action of selected compounds on the cell proliferation of the tested cancer cell lines was assessed using the MTT assay. For this testing, mitochondrial succinate dehydrogenase was used to convert yellow MTT to purple formazan in accordance with Mosmann^55^ and Ohno and Abe^56^ 72 h after the addition of selected compounds. The cells were cultured in RPMI-1640 media supplemented with 10% fetal bovine serum. In an incubator at 37 °C and 5% CO_2_, streptomycin, and penicillin (100 g ml^−1^ and 100 units per ml, respectively) were added. Cancer cells were grown in 96-well plates for two days in 5% CO_2_ (10 000 cells per well). The cell lines were cultured for 24 hours after being exposed to various doses of the investigated chemicals. DMSO content never exceeded 0.5%, which was non-toxic to the cells. In an independent experiment, antineoplastic drug cis-platin was used as a positive control. The absorbance of treated samples was measured at 570 nm.

### Fluorescence titration of DNA and BSA

ctDNA, and BSA (bovine serum albumin) and ethidium bromide (EB) were purchased from Sigma, while buffer phosphate buffered saline (PBS) was secured by Fisher BioReagents. A stock solution of ctDNA and EB or Hoechst in double distilled water was prepared in a selected buffer (10 mM PBS) that provides pH = 7.4. Before titration started, it was necessary to check the concentration of the stock DNA solution. The solution gave a ratio of UV absorbances at 260 and 280 nm (*A*_260_/*A*_280_) of *cca.* 1.8–1.9, implying that the DNA was adequately free of proteins.

DNA and EB or Hoechst concentrations were equal (100 μM). The concentration of 4d varied from 0 to 100 μM (15, 30, 45, 60, 75, and 100 μM). A series of solutions was prepared by mixing up equal volumes of DNA and EB or Hoechst, then adding an increasing volume of 4d, and finally, adding PBS to mark in a volumetric flask (10 ml). The incubation time was 24 hours. The fluorescence spectra were measured in the range 550–750 nm or 350–530 nm upon excitation at 520 or 340 nm for DNA–EB or DNA–Hoechst species, respectively. Results were analyzed using Stern–Volmer eqn (1):^57^

1 *I*_0_/*I* = 1 + *K*_SV_[Q]

A stock BSA solution (10 μM) was prepared in buffer (10 mM PBS). Serie of 4d–BSA solutions (5–50 μM) were prepared and stored in a dark place for 12 hours at room temperature. The molar ratio of 4d : BSA was varied in order 0 : 1; 1 : 1; 2.0 : 1, 3.0 : 1; 4.0 : 1; 5.0 : 1; 6.0 : 1; 7.0 : 1; 8.0 : 1; 9.0 : 1; and 10.0 : 1 in the volumetric flask of 10.0 ml. The concentration of BSA was 5.0 μM. The fluorescence spectra were measured between 300 and 500 nm upon excitation at 287 nm. Fluorescence emission titration data were evaluated utilizing eqn (2):^58^

2 log((*I*_0_ − *I*)/*I*) = log *K*_a_ + *n* log[Q]

In eqn (1) and (2), *I*_0_ and *I* are the emission intensities in the absence and presence of the quencher, then Q, *K*_SV_, *K*_a_, and *n* are related to quencher, Stern–Volmer constant, the binding constant, and the number of binding sites per BSA molecule, respectively.

## Conclusions

Several indoline-2-one (4a–h) derivatives were synthesized and analysed using NMR, IR, ESI-MS, and elemental analysis. Isolated yields of 4a–h were moderate to good (up to 81%). The presence of a heteroaromatic core (thienyl or pyridyl) influenced the yield significantly. All indolin-2-ones were tested for antibacterial activity against Gram-positive and Gram-negative bacteria as well as fungi. Gram-negative *K. pneumoniae* strains were far more sensitive than other examined bacteria. The best antibacterial activity had molecules 4f–h (MIC = 3.1 mg ml^−1^). Despite this, the examined indolin-2-ones had better antifungal than antibacterial activity. *C. albicans*, *P. italicum*, and *A. niger* were particularly sensitive after treatment with molecules that have the *m*-aminophenyl (4d) or pyridin-2-yl (4g) functions. These indolin-2-ones were chosen for investigation of their antiproliferative effect on cancer (LS174, MCF-7, PaCa-2, and A549) and normal cell lines (MRC-5). Investigated molecules possessed moderate-to-good activity against MCF-7 cell lines. Compound 4d has the lowest IC_50_ value (18.42 ± 0.45 μM) and consequently the strongest antiproliferative potential with a selectivity index ∼6. 4d presents a promising compound with good potential for dual activity (antifungal and antiproliferative). Nevertheless, the potential of indolin-2-ones (4d and 4g) as anticandidal and anticancer agents is encouraging, and their further development may lead to the discovery of new dual-active drugs for the treatment of candidiasis combined with cancer. Overall evidence also suggests that indoline-2-ones containing arylidene and *N*-allyl fragments may be useful for the development of new dual-active pharmaceuticals or even dual-active polymers.

## Author contributions

Conceptualization, writing original draft preparation and supervision S. N. A. B., and N. J.; synthesis, characterization, and binding study S. N. A. B., and N. J.; methodology and visualization H. E., T. G. A., N. A., W. A., M. A. E., N. H. A., and K. J. All authors have read and agreed to the published version of the manuscript.

## Conflicts of interest

There are no conflicts to declare.

## Supplementary Material

RA-013-D3RA04997C-s001
