# Supplementary material for: Dual activity of indolin-2-ones containing an arylidene motif: DNA and BSA interaction
Source: RSC Adv. 2023 Sep 25;13(40):28139–47. doi: 10.1039/d3ra04997c (PMC10518658; doi:10.1039/d3ra04997c)
Supplement: RA-013-D3RA04997C-s001 [file RA-013-D3RA04997C-s001.pdf]

Electronic Supplementary Material (ESI) for RSC Advances.
This journal is © The Royal Society of Chemistry 2023

RSC ADVANCES

Supplementary Information associated with the paper

Dual activity of indolin-2-ones containing an arylidene motif: DNA and BSA interaction

Syed Nasir Abbas Bukhari[1*], Tariq G. Alsahli[2], Hasan Ejaz[3], Naveed Ahmed[4], Waqas Ahmad[5], Mervat A. Elsherif[6], Nasser H. Alotaibi[7], Kashaf Junaid[8], and Nenad Janković[9*]

[1]Department of Pharmaceutical Chemistry, College of Pharmacy, Jouf University, Sakaka, Al Jouf, 72388, Saudi Arabia; sbukhari@ju.edu.sa (SNAB)

[2] Department of Pharmacology, College of Pharmacy, Jouf University, Sakaka, Al Jouf, 72388, Saudi Arabia; TGAlsahli@ju.edu.sa (TGA)

[3] Department of Clinical Laboratory Sciences, College of Applied Medical Sciences, Sakaka, Jouf Universi-ty, Al Jouf, 72388, Saudi Arabia; hetariq@ju.edu.sa (HE)

[4] Department of Pharmaceutics, College of Pharmacy, Jouf University, Sakaka, Al Jouf, 72388, Saudi Arabia; nakahmad@ju.edu.sa (NA)

[5] Discipline of Pharmaceutical Chemistry, School of Pharmaceutical Sciences, Universiti Sains Malaysia, Gelugor, 11800, Penang, Malaysia; waqas@usm.my (WA)

[6] Chemistry Department, College of Science, Jouf University, Al Jouf, 72388, Sakaka, Saudi Arabia; maelsherif@ju.edu.sa (MAE)

[7] Department of Clinical Pharmacy, College of Pharmacy, Jouf University, Sakaka 72388, Saudi Arabia; nhalotaibi@ju.edu.sa (NHA)

[8] School of Biological and Behavioural Sciences, Queen Mary University of London, London, E1 4NS, UK Email: kashaf.junaid@qmul.ac.uk (KJ)

[9] University of Kragujevac, Institute for Information of Technologies Kragujevac, Department of Science, Jovana Cvijića bb, 34000 Kragujevac; nenad.jankovic@kg.ac.rs (NJ)

*Correspondence: sbukhari@ju.edu.sa (SNAB); nenad.jankovic@kg.ac.rs (NJ)

Contents

1. Experimental data 3

2. NMR spectra 5

2.1. ^{1}H NMR spectrum of 1-allylindoline-2,3-dione **1** 6

2.2. ^{13}C NMR spectrum of 1-allylindoline-2,3-dione **1** 7

2.3. ^{1}H NMR spectrum of 1-allyl-3-(2-oxo-2-phenylethylidene)indolin-2-one **4a** 8

2.4. ^{13}C NMR spectrum of 1-allyl-3-(2-oxo-2-phenylethylidene)indolin-2-one **4a** 9

2.5. ^{1}H NMR spectrum of 1-allyl-3-(2-oxo-2-p-tolylethylidene)indolin-2-one **4b** 10

2.6. ^{13}C NMR spectrum of 1-allyl-3-(2-oxo-2-p-tolylethylidene)indolin-2-one **4b** 11

2.7. ^{1}H NMR spectrum of 1-allyl-3-(2-(4-bromophenyl)-2-oxoethylidene)indolin-2-one **4c** 12

2.8. ^{13}C NMR spectrum of 1-allyl-3-(2-(4-bromophenyl)-2-oxoethylidene)indolin-2-one **4c** 13

2.9. ^{1}H NMR spectrum of 1-allyl-3-(2-(3-aminophenyl)-2-oxoethylidene)indolin-2-one **4d** 14

2.10. ^{13}C NMR spectrum of 1-allyl-3-(2-(3-aminophenyl)-2-oxoethylidene)indolin-2-one **4d** 15

2.11. ^{1}H NMR spectrum of 1-allyl-3-(2-(3-nitrophenyl)-2-oxoethylidene)indolin-2-one **4e** 16

2.12. ^{13}C NMR spectrum 1-allyl-3-(2-(3-nitrophenyl)-2-oxoethylidene)indolin-2-one **4e** 17

2.13. ^{1}H NMR spectrum of 1-allyl-3-(2-(3-methoxyphenyl)-2-oxoethylidene)indolin-2-one **4f** 18

2.14. ^{13}C NMR spectrum of 1-allyl-3-(2-(3-methoxyphenyl)-2-oxoethylidene)indolin-2-one **4f** 19

2.15. ^{1}H NMR spectrum of 1-allyl-3-(2-oxo-2-(pyridin-2-yl)ethylidene)indolin-2-one **4g** 20

2.16. ^{13}C NMR spectrum of 1-allyl-3-(2-oxo-2-(pyridin-2-yl)ethylidene)indolin-2-one **4g** 21

2.17. ^{1}H NMR spectrum of 1-allyl-3-(2-oxo-2-(thiophen-2-yl)ethylidene)indolin-2-one **4h** 22

2.18. ^{13}C NMR spectrum of 1-allyl-3-(2-oxo-2-(thiophen-2-yl)ethylidene)indolin-2-one **4h** 23

3. MS spectra of 4a-h 24

4. IR spectra of 4a-h 29

1. Experimental data

1-allylindoline-2,3-dione 1

Red solid; Yield: 96%; Mp = 95 °C; ^{1}H NMR (200 MHz, DMSO-d_6) δ 7.68-7.53 (m, 2H, CH_{Ar}), 7.16 -7.03 (m, 2H, CH_{Ar}), 5.96 – 5.77 (m, 1H, =CH), 5.39-5.16 (m, 2H, =CH_2), 4.31 (dt, *J* = 4.9, 1.7 Hz, 2H, N-CH_2) ppm; ^{13}C NMR (50 MHz, DMSO-d_6) δ 183.2, 157.9, 150.5, 138.1, 131.4, 124.5, 123.3, 117.5, 111.2, 41.9 ppm.

1-allyl-3-(2-oxo-2-phenylethylidene)indolin-2-one 4a

Orange solid; Yield: 54 % (1,06 g); Mp = 85 °C; IR ν 3434, 1706, 1658, 1619, 1600, 1464, 1346, 1225 cm^{-1}; ^{1}H NMR (200 MHz, DMSO-d_6) δ 8.11-8.03 (m, 3H, CH_{Ar}), 7.81 (s, 1H, =CH), 7.77-7.68 (m, 1H, CH_{Ar}), 7.63-7.55 (m, 2H, CH_{Ar}), 7.44-7.35 (m, 1H, CH_{Ar}), 7.04-6.97 (m, 2H, CH_{Ar}), 5.97-5.78 (m, 1H, =CH_{allyl}), 5.24-5.14 (m, 2H, =CH_2), 4.39-4.37 (m, 2H, N-CH_2) ppm; ^{13}C NMR (50 MHz, DMSO-d_6) δ 191.3, 166.5, 144.9, 136.9, 135.0, 134.2, 132.7, 131.8, 129.2, 128.7 (2C), 127.1 (2C), 126.4, 122.4, 119.4, 117.1, 109.8, 41.9 ppm; ESI-MS (*m/z*): $[M+H]^+$ = 290; Cacld. for $C_{19}H_{15}NO_2$: C 78.87; H 5.23; N 4.84; Found: C 78.64; H 5.12; N 4.69.

1-allyl-3-(2-oxo-2-*p*-tolylethylidene)indolin-2-one 4b

Orange solid; Yield: 49 % (1,13 g); Mp = 90-92 °C; IR ν 3434, 1714, 1657, 1615, 1605, 1465, 1360, 1348 cm^{-1}; ^{1}H NMR (200 MHz, DMSO-d_6) δ 8.01-7.95 (m, 3H, CH_{Ar}), 7.79 (s, 1H, =CH), 7.41-7.32 (m, 3H, CH_{Ar}), 7.03-6.96 (m, 2H, CH_{Ar}), 6.01-5.78 (s, 1H, =CH_{allyl}), 5.23-5.14 (m, 2H, CH_2), 4.37 (d, *J* = 5.0 Hz, 2H, N-CH_2), 2.39 (s, 3H, CH_3) ppm; ^{13}C NMR (50 MHz, DMSO-d_6) δ 190.8, 166.6, 144.9, 144.8, 134.7, 134.5, 132.6, 131.8, 129.8 (2C), 128.8 (2C), 127.4, 126.3, 122.3, 119.4, 117.1, 109.7, 41.9, 21.5 ppm; ESI-MS (*m/z*): $[M+H]^+$ = 304; Cacld. for $C_{20}H_{17}NO_2$: C 79.19; H 5.65; N 4.62; Found: C 79.02; H 5.48; N 4.50.

1-allyl-3-(2-(4-bromophenyl)-2-oxoethylidene)indolin-2-one 4c

Red solid; Yield: 80 % (2,21 g); Mp = 98 °C; IR ν 3432, 1716, 1665, 1616, 1596, 1466 cm^{-1}; ^{1}H NMR (200 MHz, DMSO-d_6) δ 8.07-7.97 (m, 3H, CH_{Ar}), 7.97-7.70 (m, 3H, =CH + CH_{Ar}), 7.45-7.35 (m, 1H, CH_{Ar}), 7.06-6.98 (m, 2H, CH_{Ar}), 6.02-5.78 (s, 1H, =CH_{allyl}), 5.23-5.14 (m, 2H, CH_2), 4.37 (d, *J* = 5.0 Hz, 2H, N-CH_2) ppm; ^{13}C NMR (50 MHz, DMSO-d_6) δ 190.3, 166.5, 145.1, 136.0, 135.5, 132.9 (2C), 132.2 (2C), 131.8, 130.6, 128.4, 126.6, 126.4, 122.4, 119.4, 117.2, 109.8, 41.9 ppm; ESI-MS (*m/z*): $[M]^+$ = 368; Cacld. for $C_{19}H_{14}BrNO_2$: C 61.97; H 3.83; N 3.80; Found: C 61.70; H 3.64; N 3.72.

1-allyl-3-(2-(3-aminophenyl)-2-oxoethylidene)indolin-2-one 4d

Dark red crystals; Yield: 43 % (1,15 g); Mp = 168 °C; IR ν 3435, 2803, 2752, 2572, 1710, 1657, 1614, 1599, 1466, 1256 cm^{-1}; ^{1}H NMR (200 MHz, DMSO-d_6) δ 8.07 (d, *J* = 7.4 Hz, 1H, CH_{Ar}), 8.02-7.96 (m, 2H, CH_{Ar}), 7.77 (s, 1H, =CH), 7.66-7.60 (m, 2H, CH_{Ar}), 7.45-7.37 (m, 1H, CH_{Ar}), 7.06-6.98 (m, 2H, CH_{Ar}), 5.96-5.78 (m, 1H, =CH_{allyl}), 5.22-5.13 (m, 2H, =CH_2), 4.38 (d, *J* = 5.0 Hz, 2H, N-CH_2) ppm; ^{13}C NMR (50 MHz, DMSO-d_6) δ 190.4, 166.6, 145.1, 138.1, 136.0, 135.6, 133.0, 131.8, 130.6, 127.2, 126.6, 126.5, 126.2, 122.5, 121.4, 119.4, 117.2, 109.9, 41.9 ppm; ESI-MS (*m/z*): $[M+H]^+$ = 305; Cacld. for $C_{19}H_{16}N_2O_2$: C 74.98; H 5.30; N 9.20; Found: C 74.82; H 5.15; N 9.28.

1-allyl-3-(2-(3-nitrophenyl)-2-oxoethylidene)indolin-2-one 4e

Brown solid; Yield: 81 % (2,10 g); Mp = 130 °C; IR ν 3428, 1711, 1657, 1609, 1530, 1467, 1346 cm^{-1}; ^{1}H NMR (200 MHz, DMSO-d_6) δ 8.73-8.71 (m, 1H, CH_{Ar}), 8.51-8.48 (m, 2H, CH_{Ar}), 8.16 (d, *J* = 7.4 Hz, 1H, CH_{Ar}), 7.92-7.82 (m, 2H, =CH + CH_{Ar}), 7.48-7.39 (m, 1H, CH_{Ar}), 7.08-6.99 (m, 2H, CH_{Ar}), 6.04-5.79 (m, 1H, =CH_{allyl}), 5.24-5.15 (m, 2H, =CH_2), 4.40-4.38 (m, 2H, N-CH_2) ppm; ^{13}C NMR (50 MHz, DMSO-d_6) δ 189.2, 166.5, 148.2, 145.4, 138.2, 136.5, 134.8, 133.4, 131.7, 131.0, 128.1,

127.0, 125.4, 122.9, 122.5, 119.3, 117.2, 109.8, 41.9 ppm; ESI-MS (*m/z*): $[M+H]^+$ = 335; Cacld. for $C_{19}H_{14}N_2O_4$: C 68.26; H 4.22; N 8.38; Found: C 68.10; H 4.20; N 8.25.

1-allyl-3-(2-(3-methoxyphenyl)-2-oxoethylidene)indolin-2-one 4f

Orange amorphous solid; Yield: 64 % (1,31 g); Mp = 80 °C; IR ν 3434, 1715, 1657, 1620, 1591, 1462, 1354, 1262 cm^{-1}; ^1H NMR (200 MHz, DMSO-d_6) δ 8.03 (d, *J* = 7.3 Hz, 1H, CH_{Ar}), 7.78 (s, 1H, =CH), 7.69-7.63 (m, 1H, CH_{Ar}), 7.54-7.26 (m, 4H, CH_{Ar}), 7.05-6.97 (m, 2H, CH_{Ar}), 6.02-5.76 (m, 1H, $=CH_{allyl}$), 5.23-5.14 (m, 2H, $=CH_2$), 4.37 (d, *J* = 5.0 Hz, 2H, N-CH_2), 3.84 (s, 3H, OCH_3) ppm; ^{13}C NMR (50 MHz, DMSO-d_6) δ 191.0, 166.6, 159.7, 145.0, 138.3, 135.1, 132.8, 131.8, 130.4, 127.1, 126.4, 122.4, 121.5, 120.4, 119.4, 117.2, 112.6, 109.8, 55.6, 41.9 ppm; ESI-MS (*m/z*): $[M+H]^+$ = 320; Cacld. for $C_{20}H_{17}NO_3$: C 75.22; H 5.37; N 4.39; Found: C 75.12; H 5.26; N 4.45.

1-allyl-3-(2-oxo-2-(pyridin-2-yl)ethylidene)indolin-2-one 4g

Dark red crystals; Yield: 27 % (0,60 g); Mp = 118-120 °C; IR ν 3434, 1704, 1668, 1619, 1597, 1466, 1363, 1226 cm^{-1}; ^1H NMR (200 MHz, DMSO-d_6) δ 8.82-8.87 (m, 1H, CH_{Ar}), 8.55-8.51 (m, 1H, CH_{Ar}), 8.45 (s, 1H, =CH), 8.14-8.07 (m, 2H, CH_{Ar}), 7.74-7.72 (m, 1H, CH_{Ar}), 7.43-7.39 (m, 1H, CH_{Ar}), 7.06-6.96 (m, 2H, CH_{Ar}), 5.96-5.77 (m, 1H, $=CH_{allyl}$), 5.22-5.13 (m, 2H, $=CH_2$), 4.38-4.35 (m, 2H, N-CH_2) ppm; ^{13}C NMR (50 MHz, DMSO-d_6) δ 190.0, 166.9, 153.4, 149.3, 145.5, 138.0, 136.5, 133.4, 131.8, 128.2, 127.6, 124.9, 122.6, 122.5, 119.7, 117.1, 109.7, 41.9 ppm; ESI-MS (*m/z*): $[M+H]^+$ = 291; Cacld. for $C_{18}H_{14}N_2O_2$: C 74.47; H 4.86; N 9.65; Found: C 74.28; H 4.71; N 9.70.

1-allyl-3-(2-oxo-2-(thiophen-2-yl)ethylidene)indolin-2-one 4h

Red solid; Yield: 27 % (0,64 g); Mp = 119-121 °C; IR ν 3428, 1709, 1643, 1613, 1464, 1413, 1352 cm^{-1}; ^1H NMR (200 MHz, DMSO-d_6) δ 8.33 (d, *J* = 7.8 Hz, 1H, CH_{Ar}), 8.18-8.13 (m, 2H, CH_{Ar}), 7.74 (s, 1H, =CH), 7.43 (td, *J* = 7.8, 1.3 Hz, 1H, CH_{Ar}), 7.32 (dd, *J* = 4.9, 3.9 Hz, 1H, CH_{Ar}), 7.09-6.98 (m, 2H, CH_{Ar}), 5.97-5.78 (m, 1H, $=CH_{allyl}$), 5.23-5.13 (m, 2H, $=CH_2$), 4.40-4.36 (m, 2H, N-CH_2) ppm; ^{13}C NMR (50 MHz, DMSO-d_6) δ 182.6, 166.6, 145.2, 144.9, 137.0, 135.8, 134.7, 133.1, 131.8, 129.4, 127.4, 125.5, 122.4, 119.5, 117.1, 109.7, 41.9 ppm; ESI-MS (*m/z*): $[M+H]^+$ = 296; Cacld. for $C_{17}H_{14}NO_2S$: C 68.90; H 4.65; N 4.73; Found: C 68.82; H 4.57; N 4.63.

2. NMR spectra

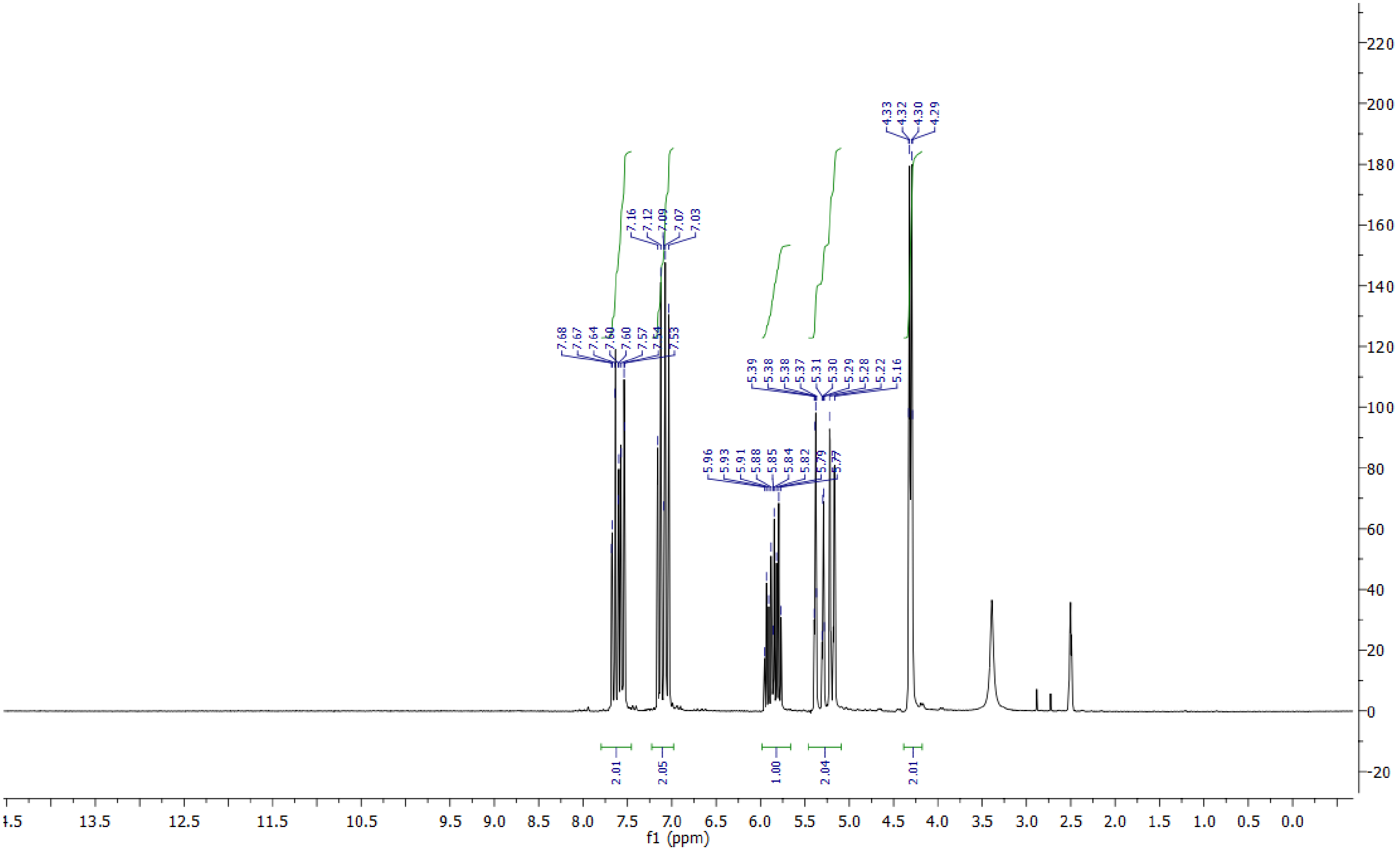

Figure S1. ^{1}H NMR spectrum of **1**

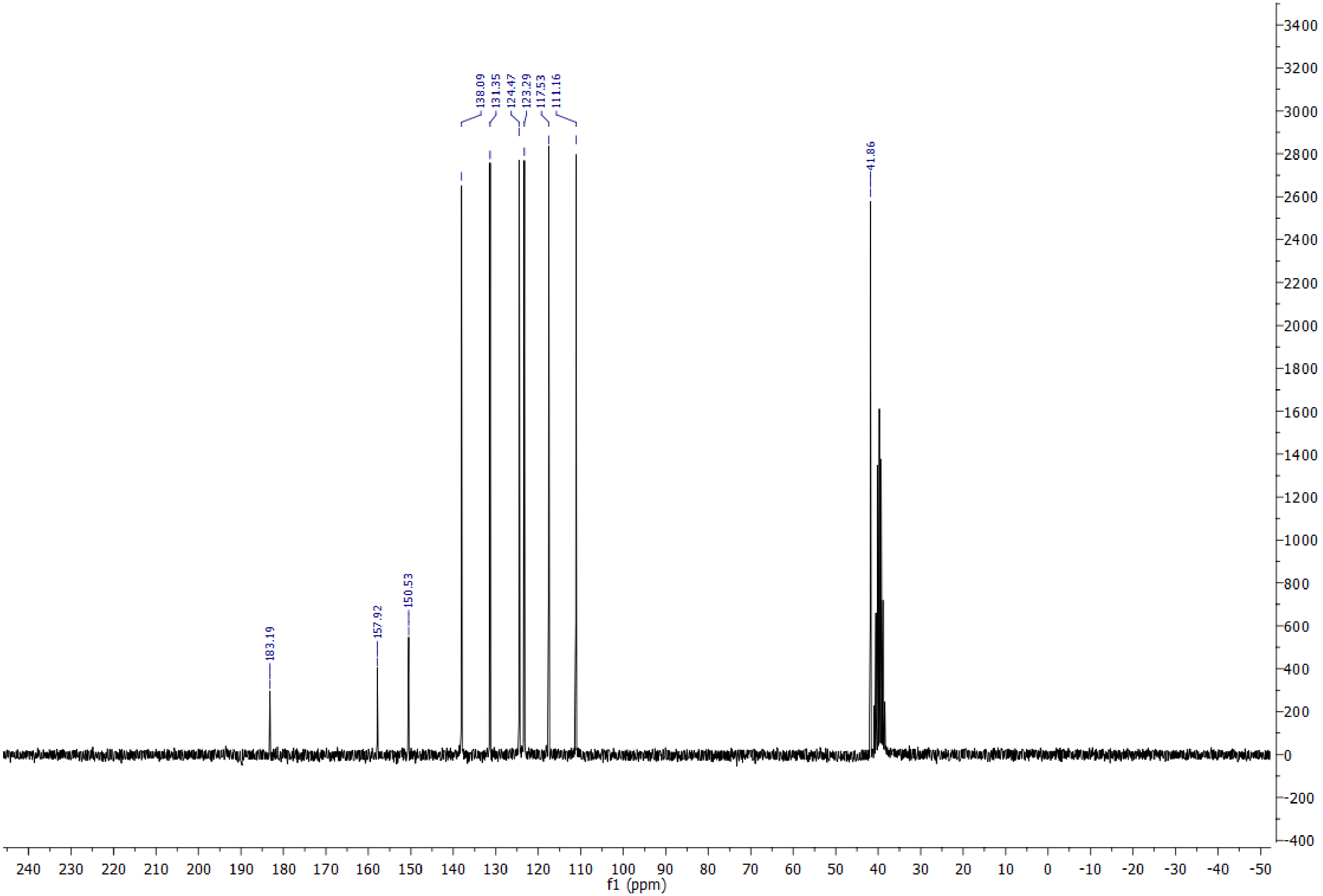

Figure S2. ^{13}C NMR spectrum of **1**

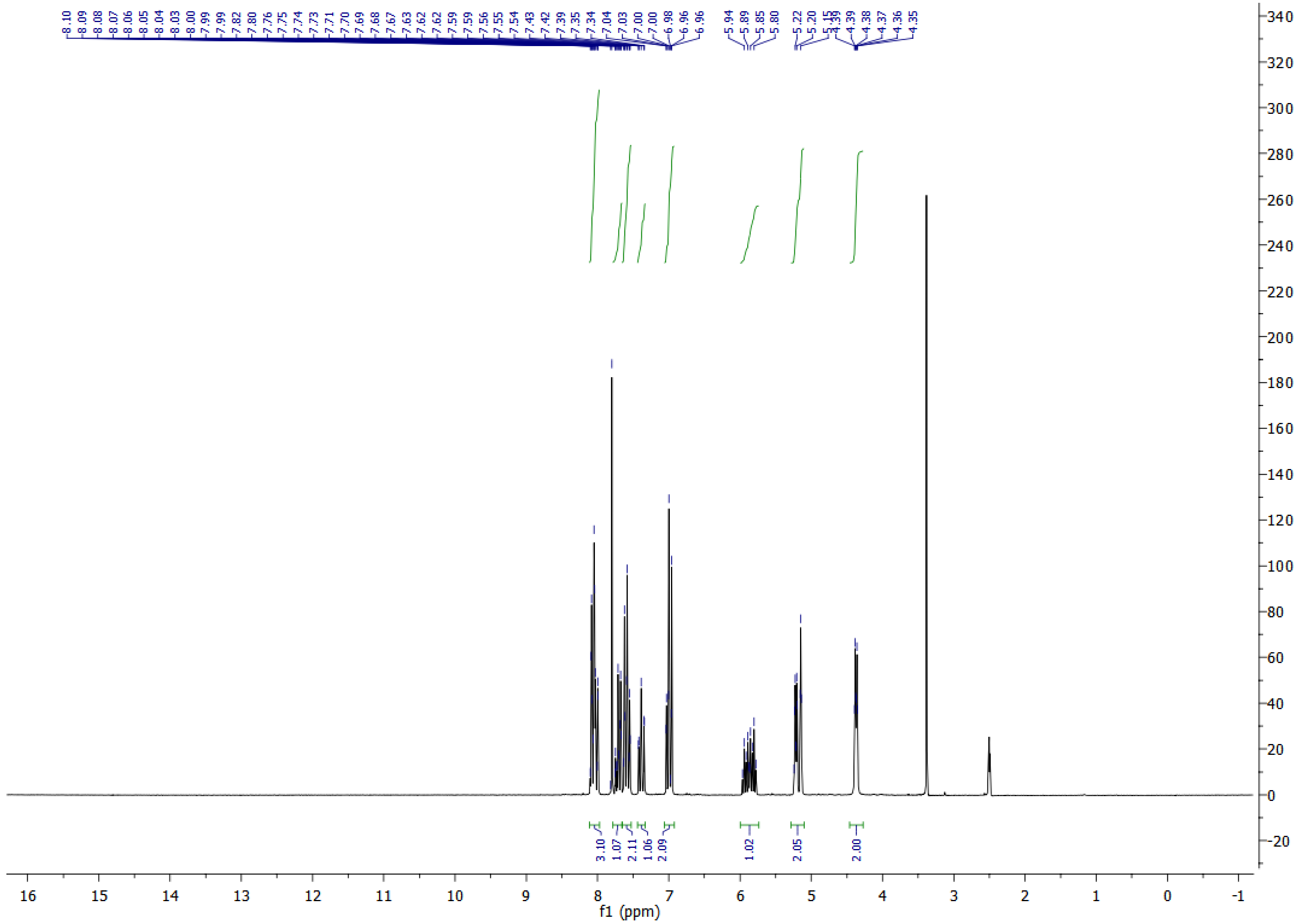

Figure S3. ^{1}H NMR spectrum of **4a**

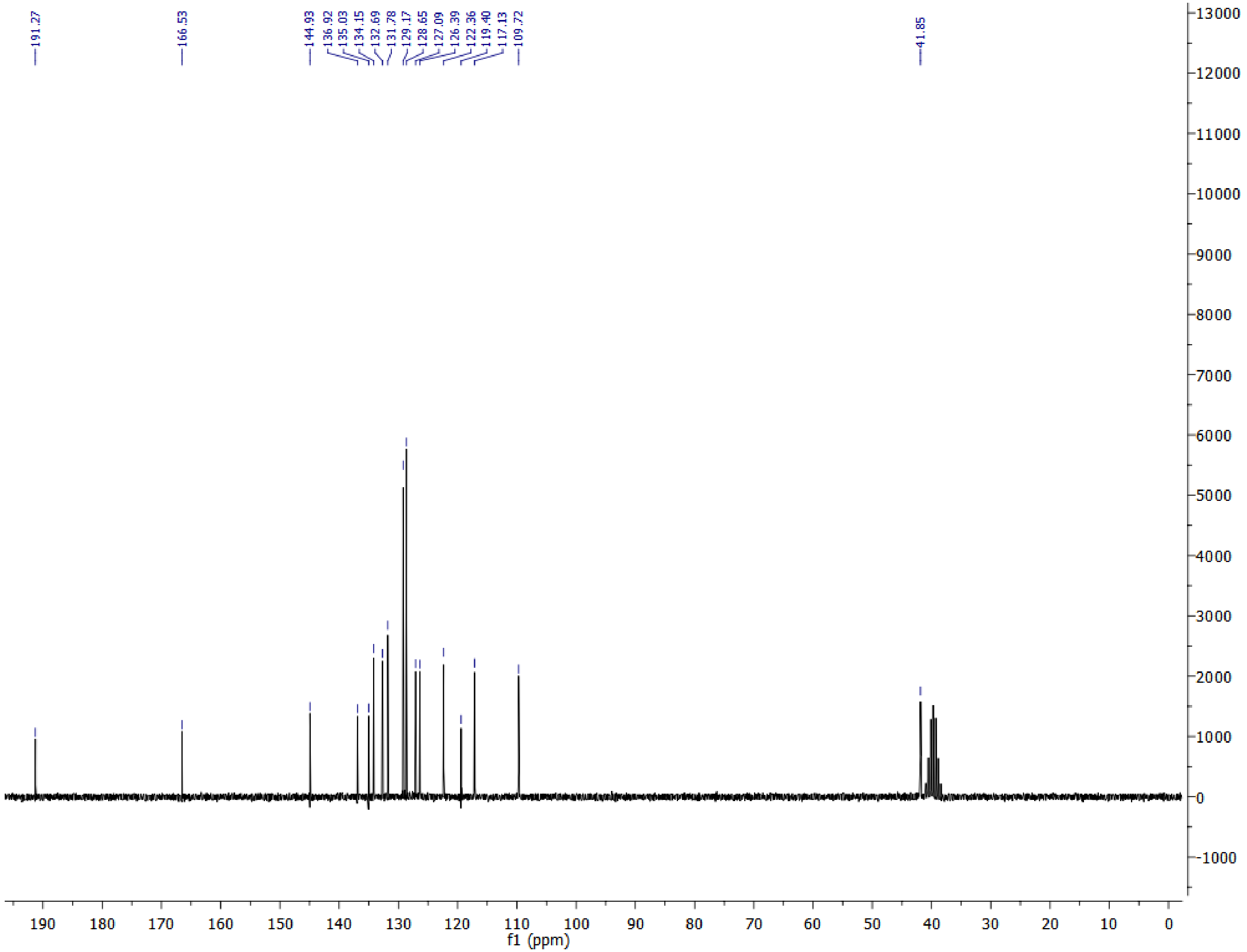

Figure S4. ^{13}C NMR spectrum of **4a**

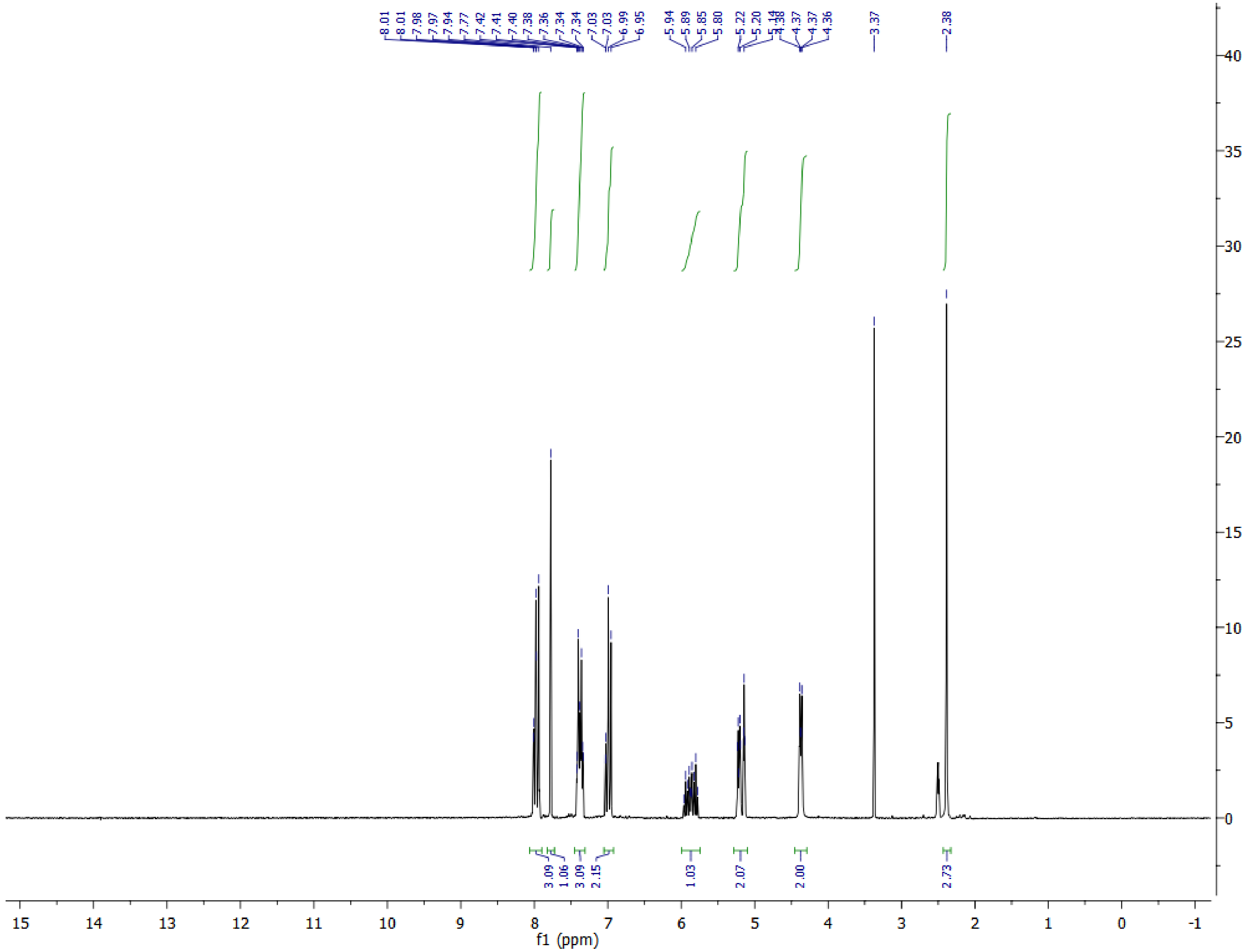

Figure S5. ^{1}H NMR spectrum of **4b**

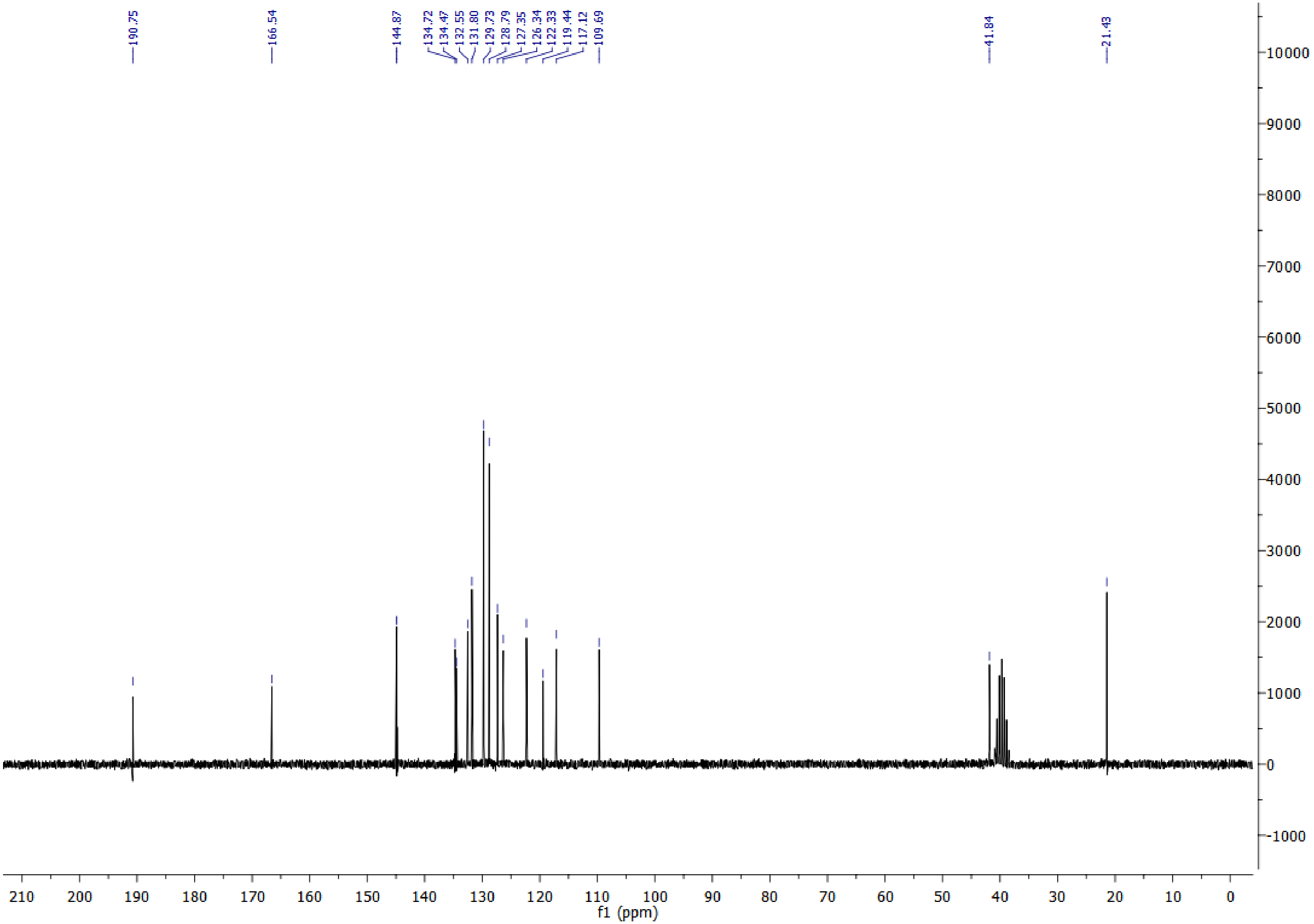

Figure S6. ^{13}C NMR spectrum of **4b**

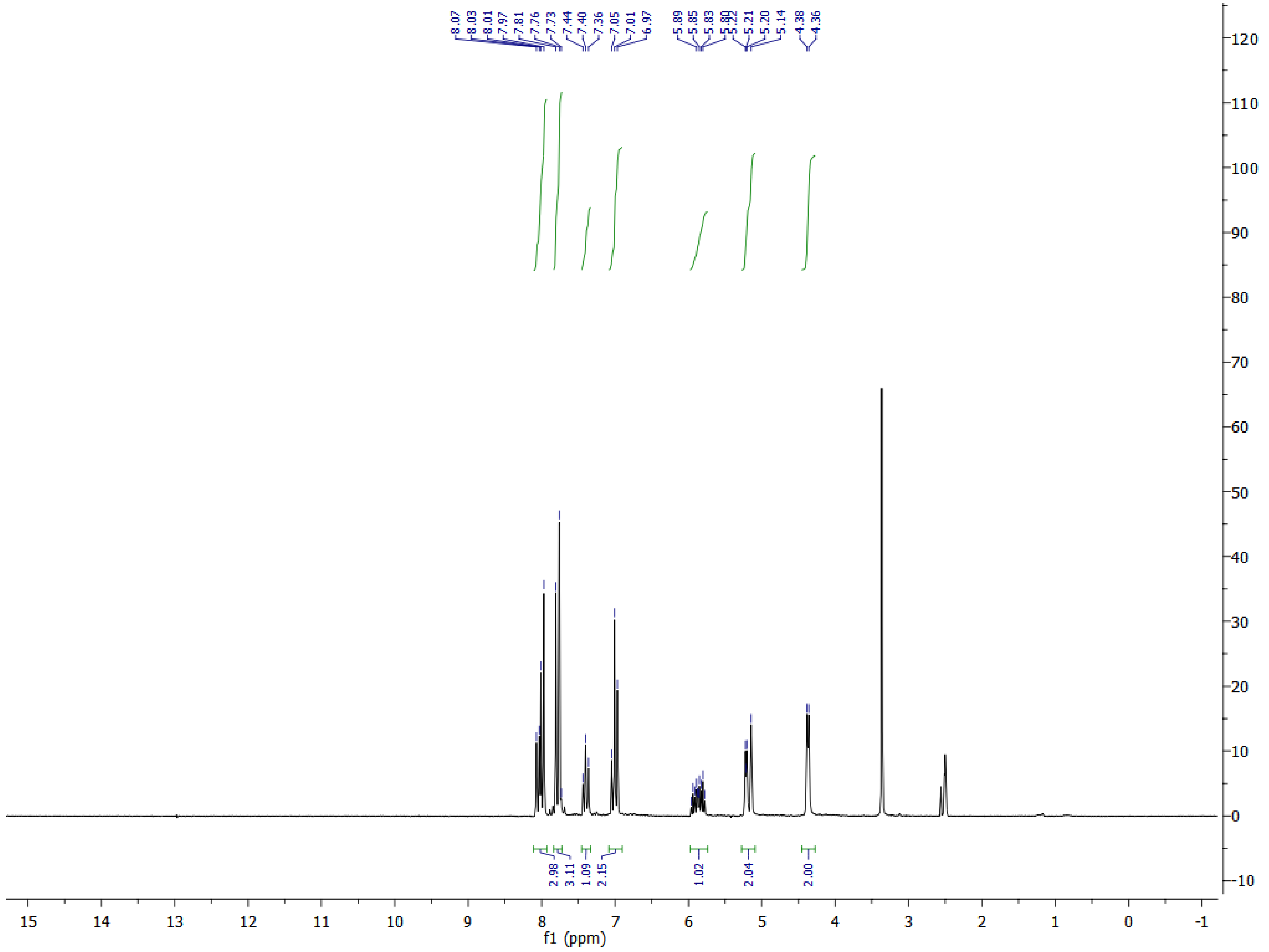

Figure S7. ^{1}H NMR spectrum of **4c** (10 mg dissolved in 600 µL DMSO-d_6)

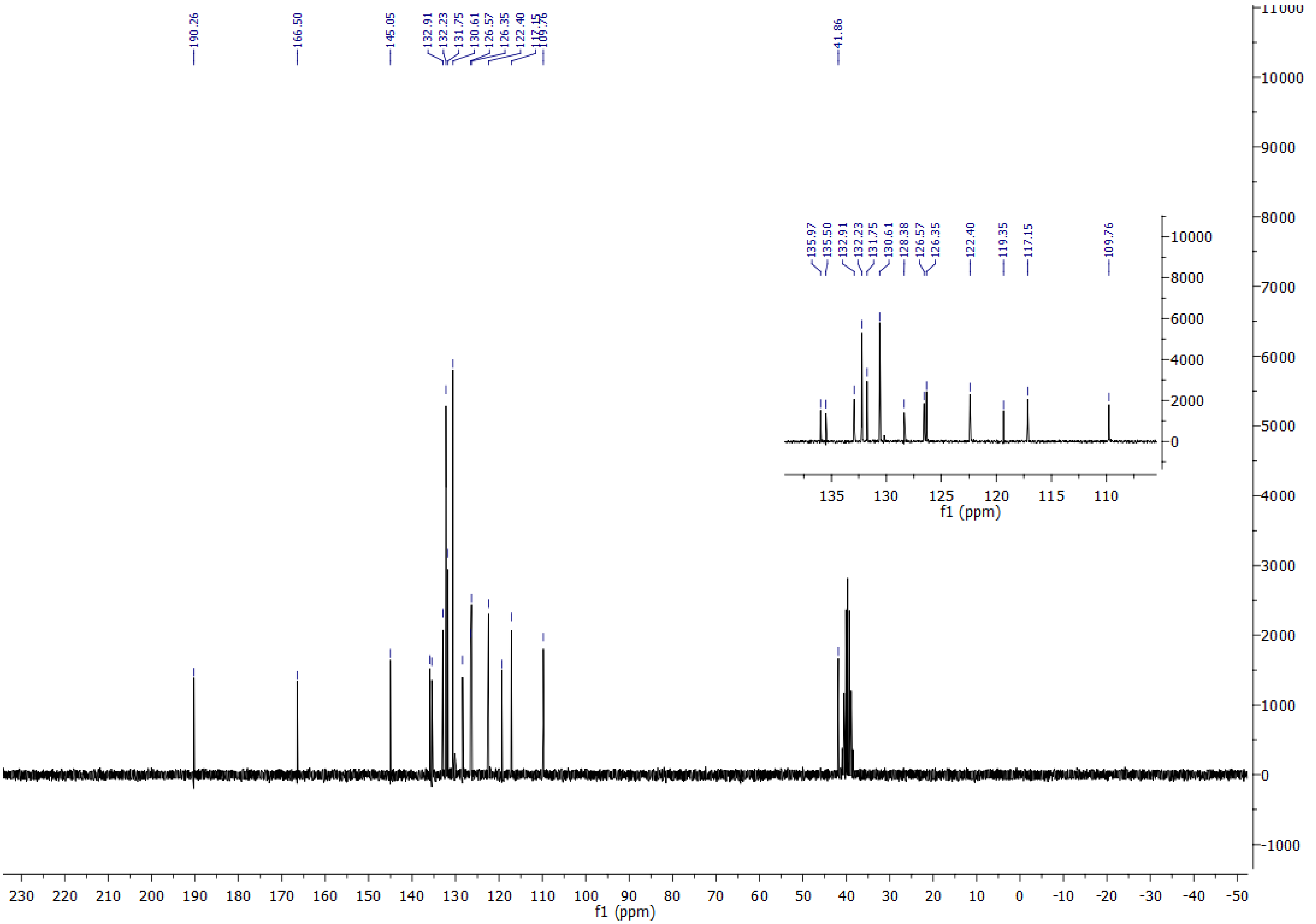

Figure S8. ^{13}C NMR spectrum of **4c**

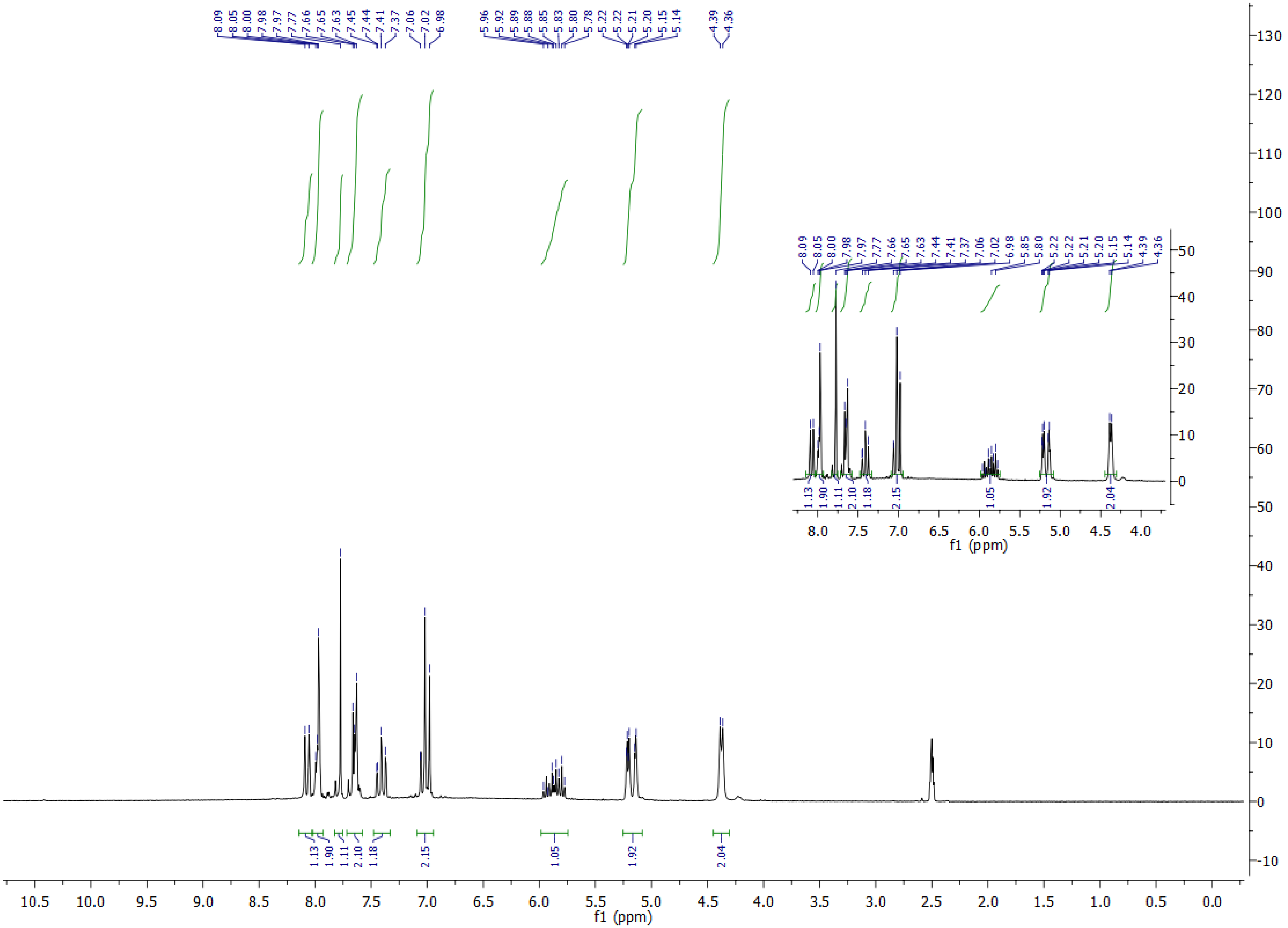

Figure S9. ^{1}H NMR spectrum of **4d**

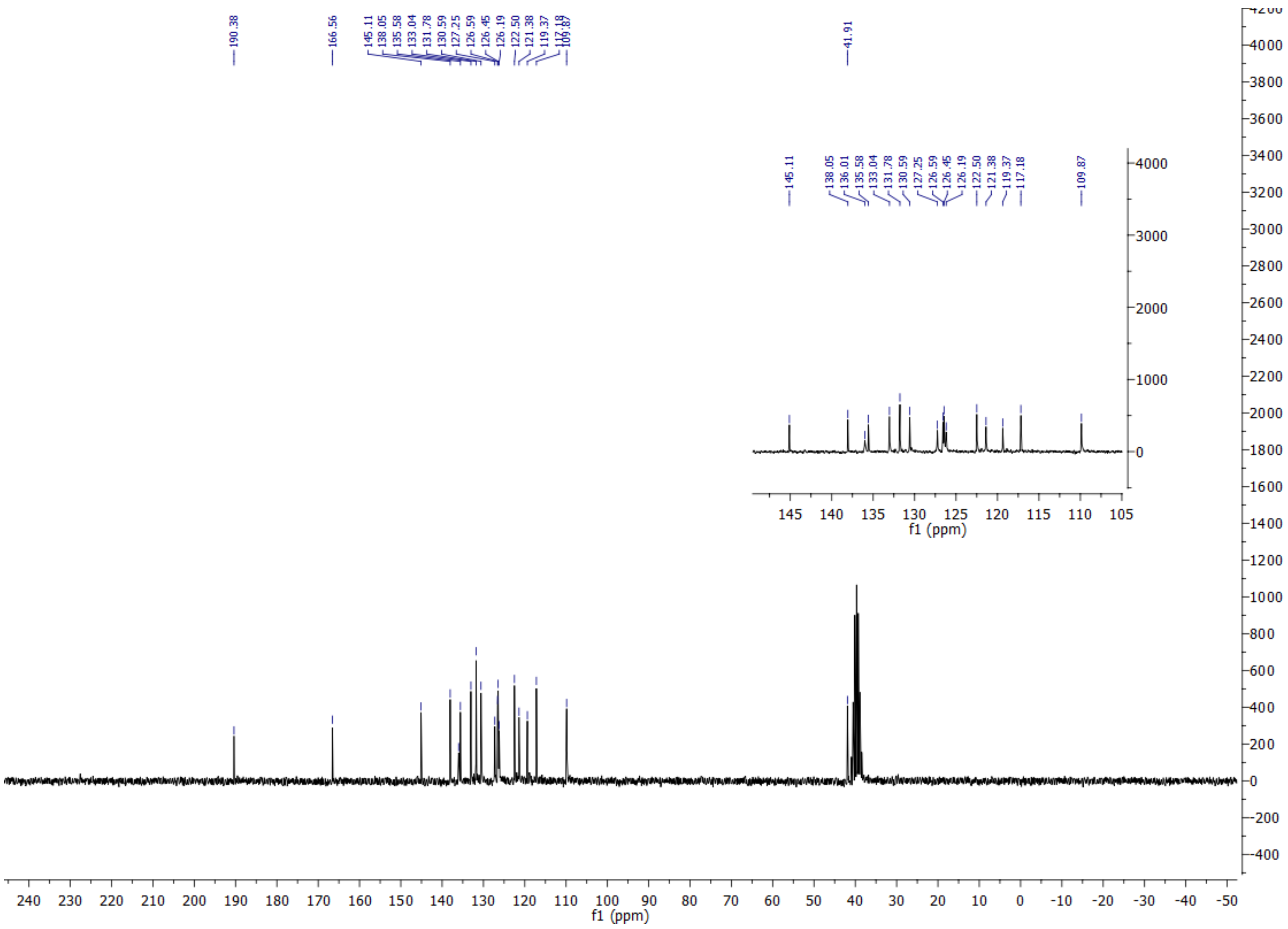

Figure S10. ^{13}C NMR spectrum of **4d**

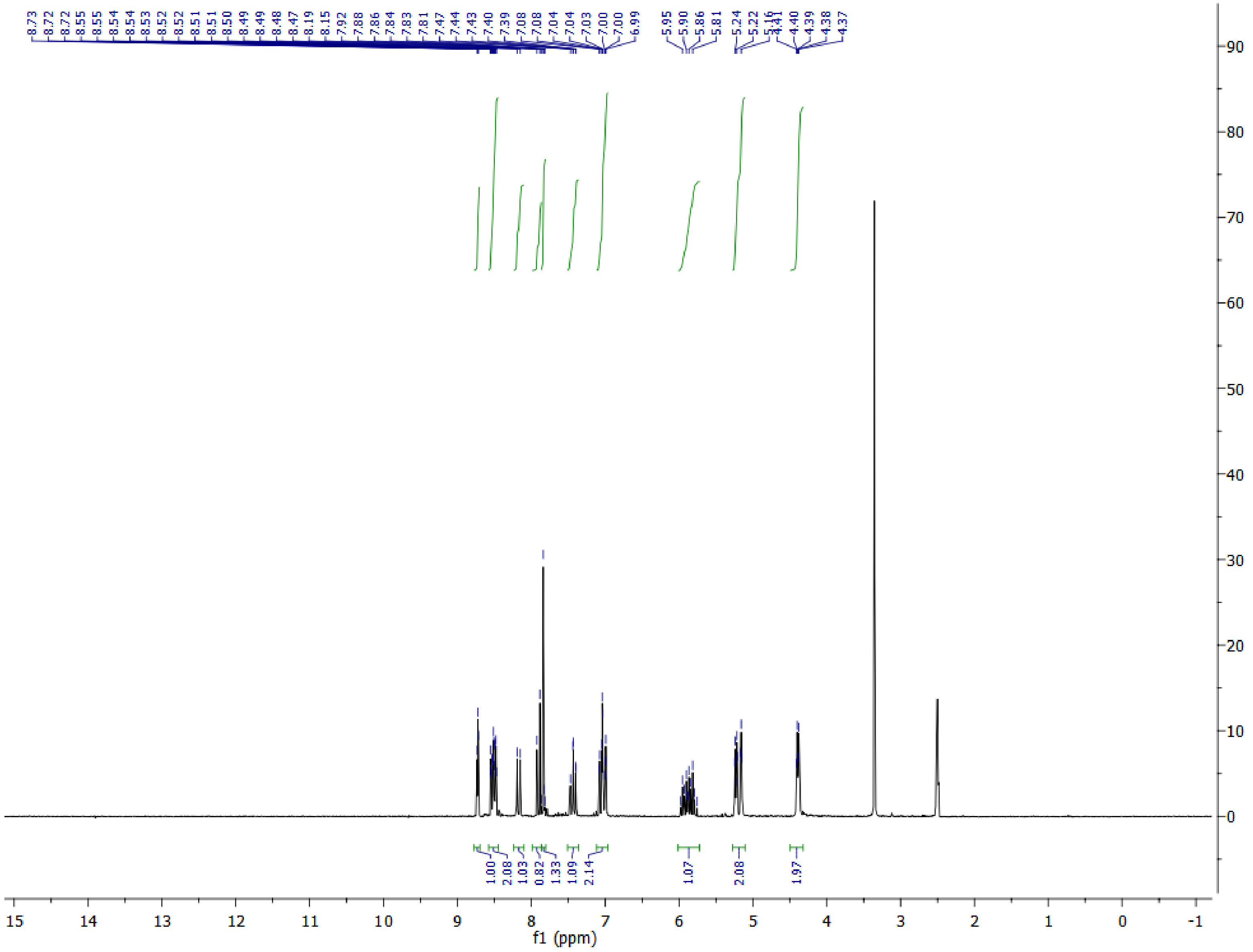

Figure S11. ^{1}H NMR spectrum of **4e** (10 mg of **4e** dissolved in 600 µL DMSO-d_6)

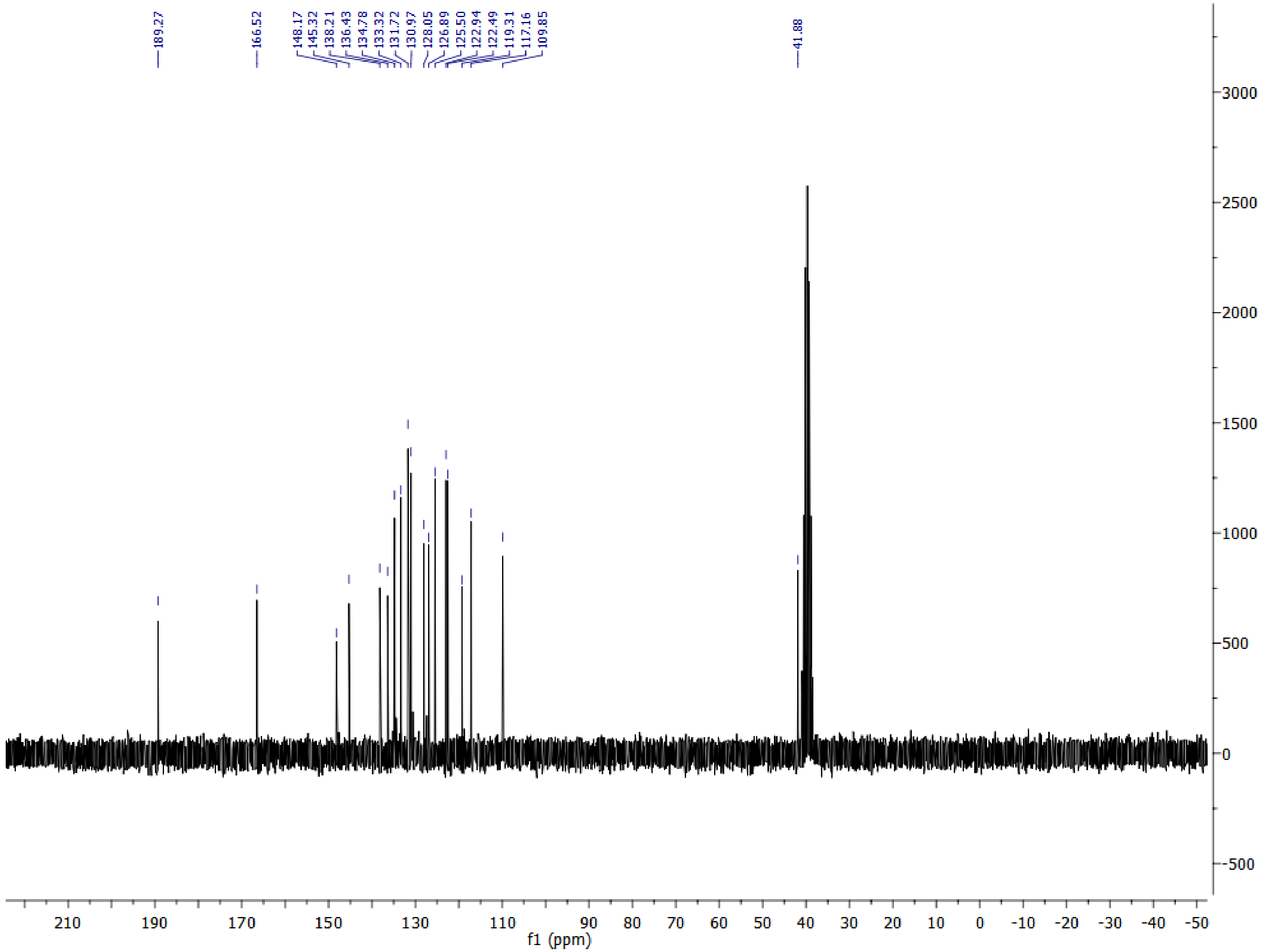

Figure S12. ^{13}C NMR spectrum of **4e**

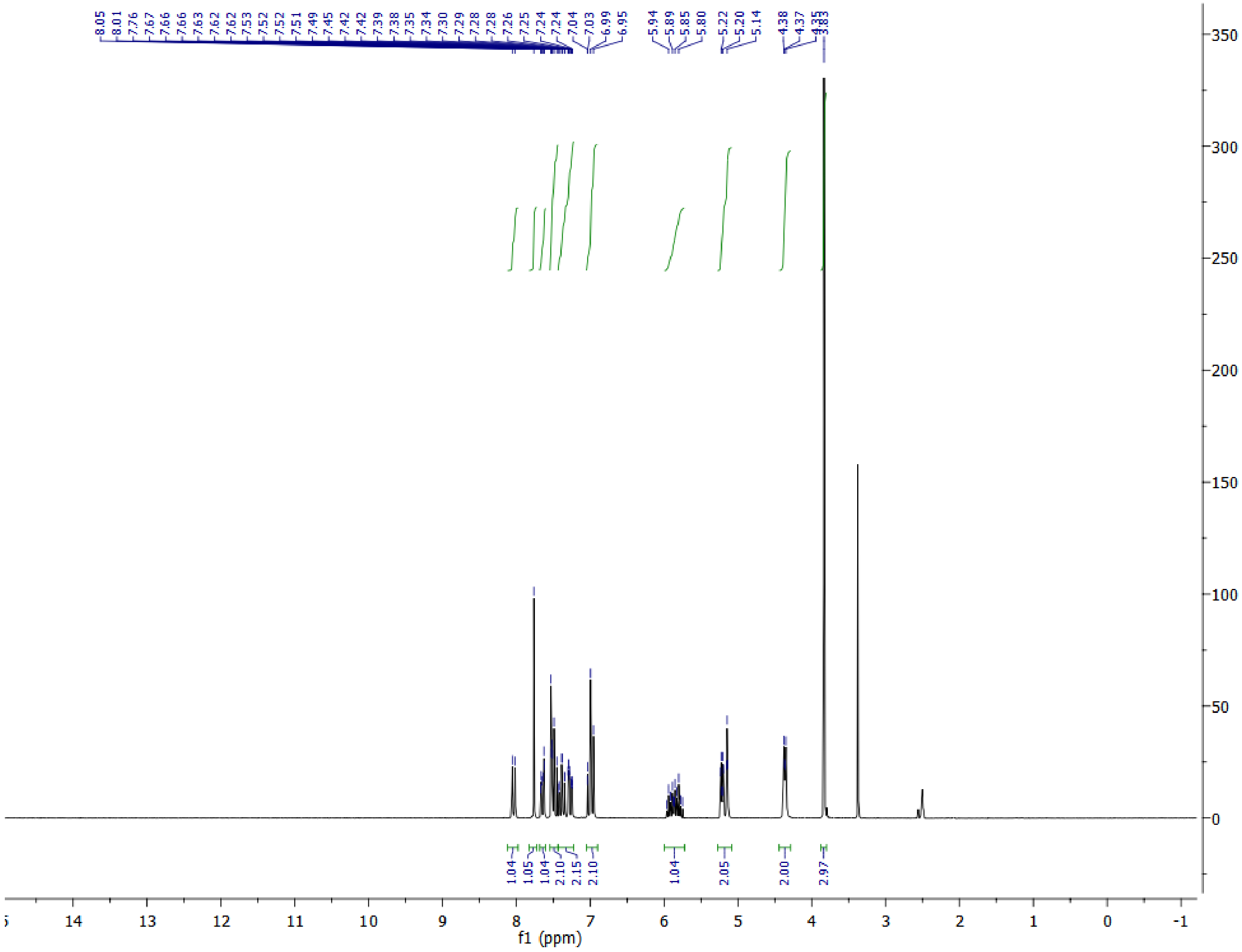

Figure S13. ^{1}H NMR spectrum of **4f**

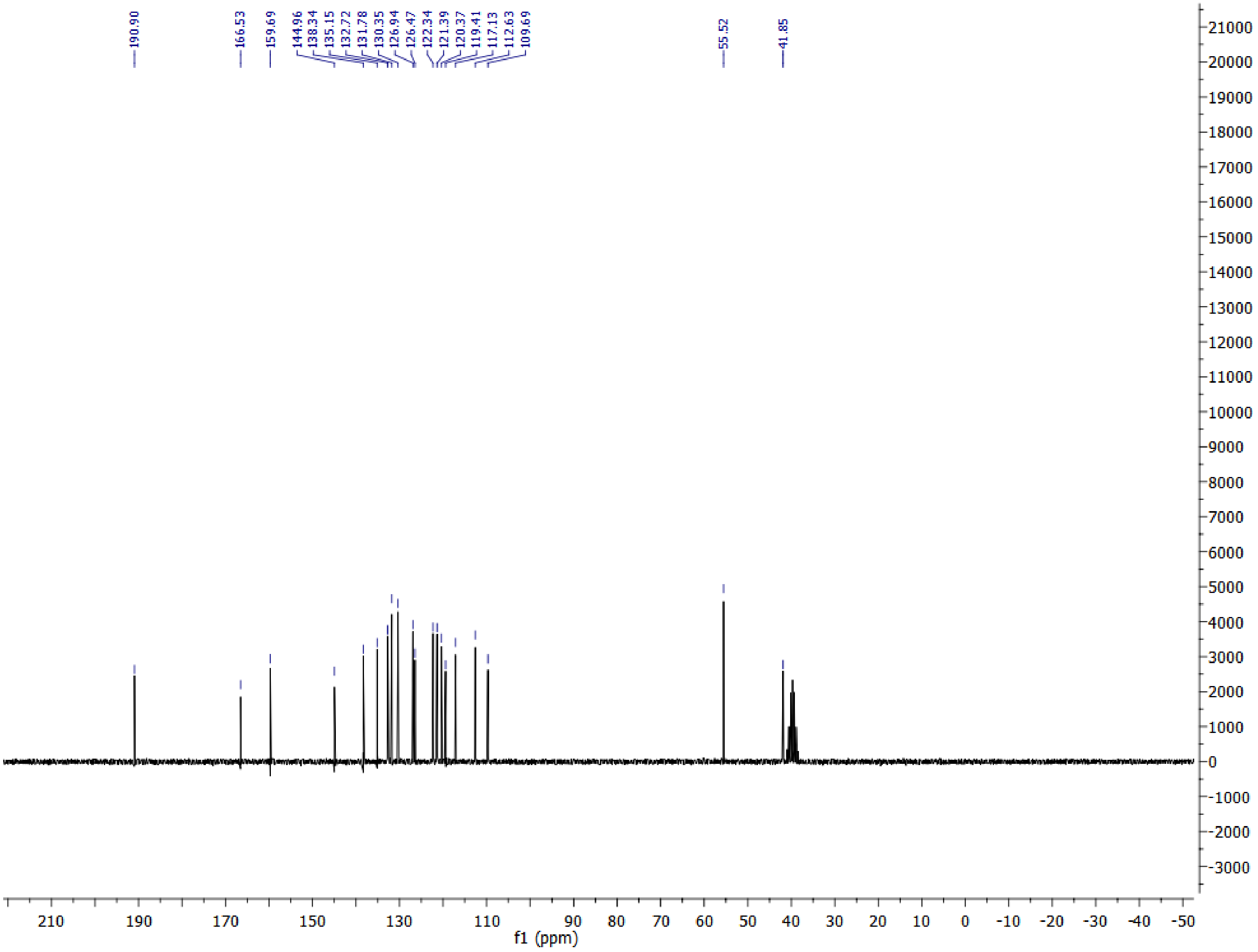

Figure S14. ^{13}C NMR spectrum of **4f**

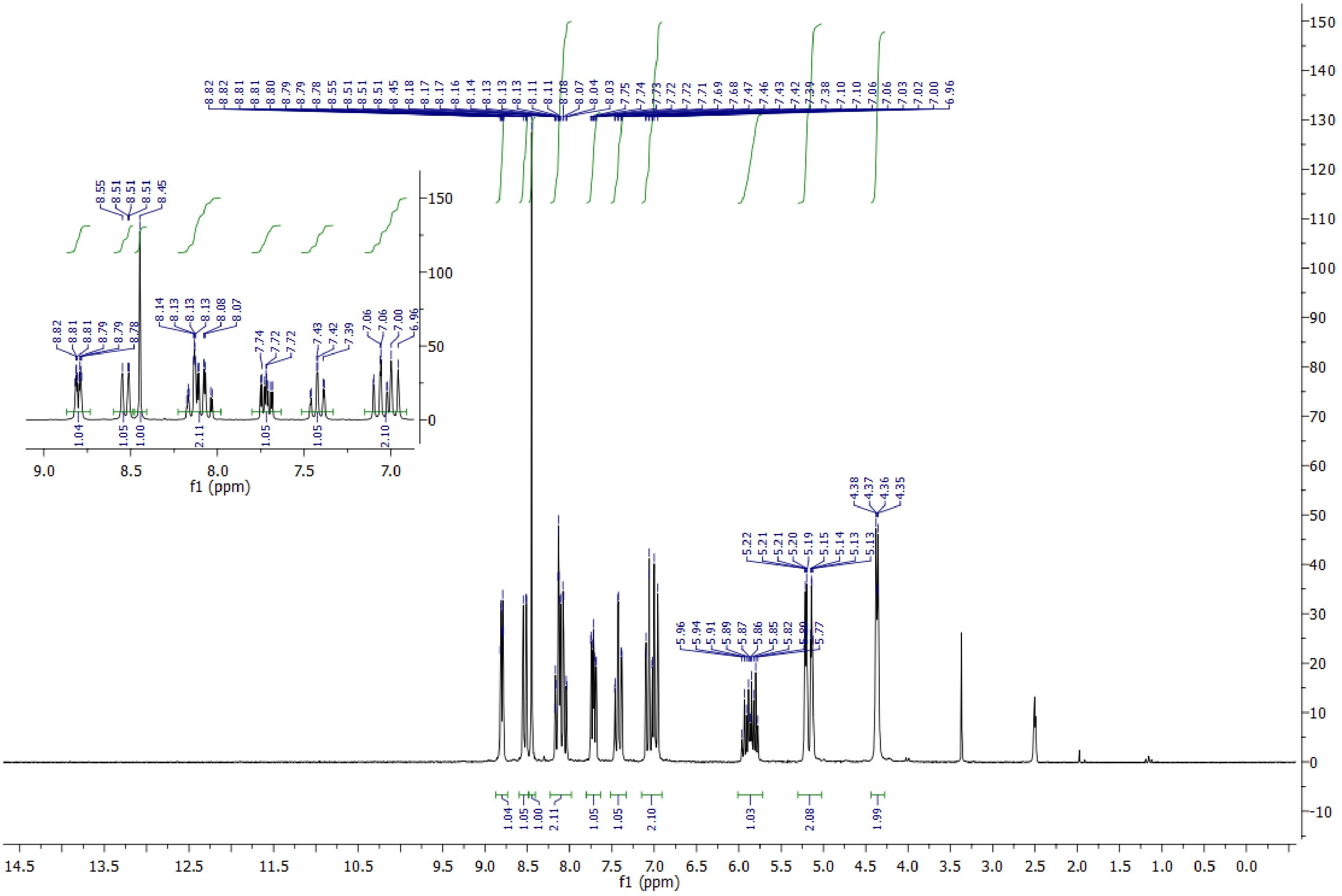

Figure S15. ^{1}H NMR spectrum of **4g**

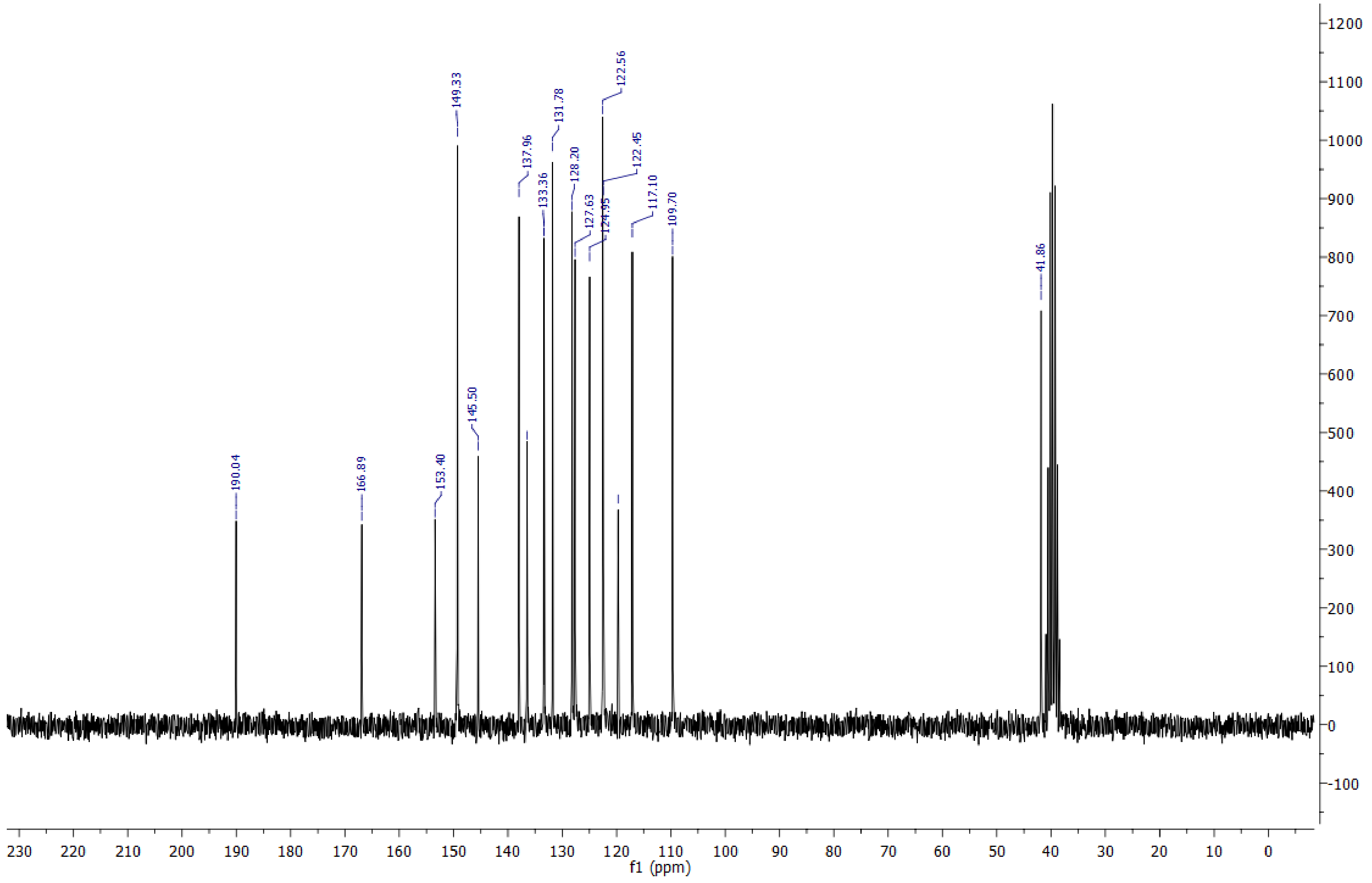

Figure S16. ^{13}C NMR spectrum of **4g**

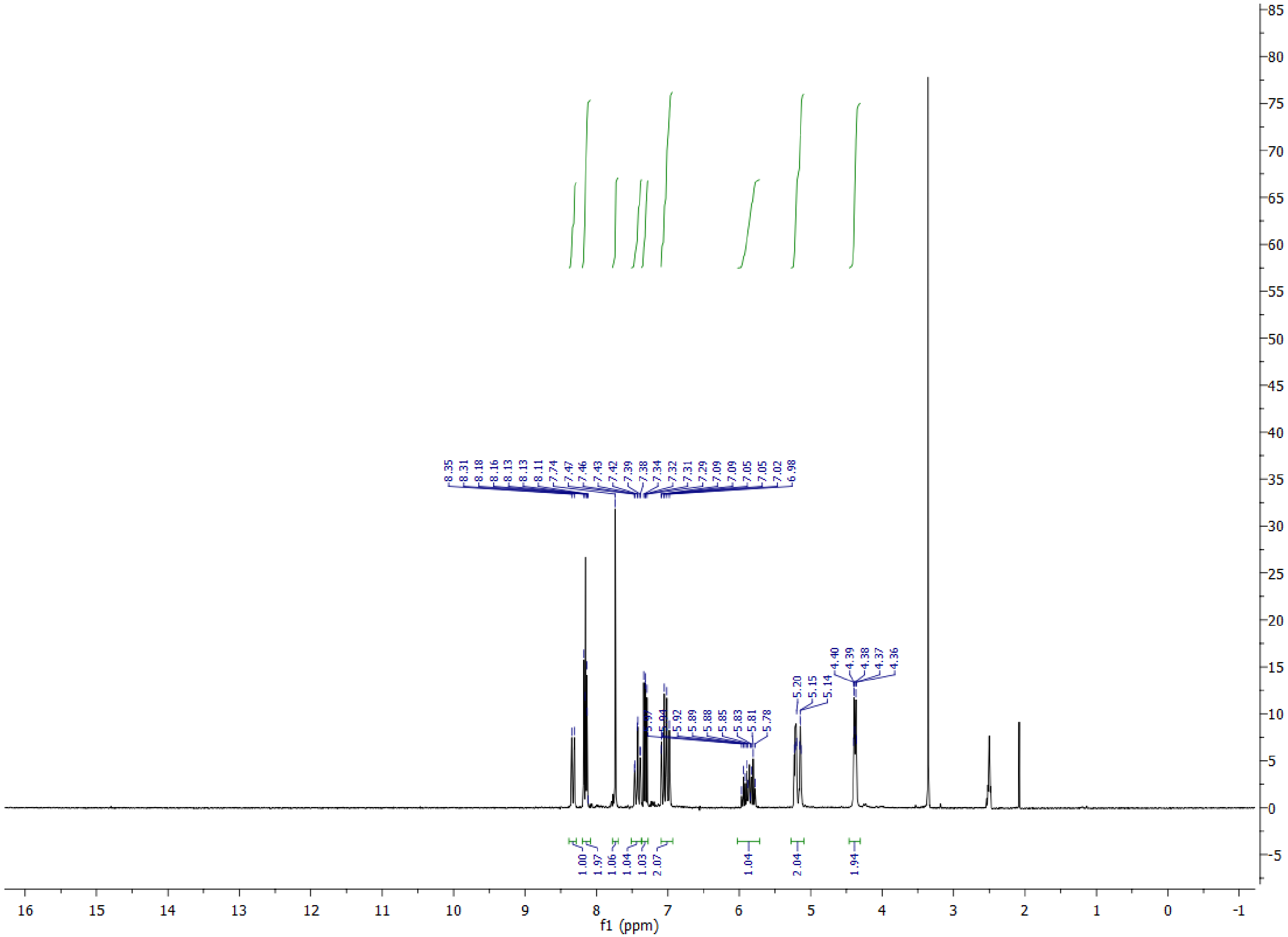

Figure S17. ^{1}H NMR spectrum of **4h** (residual solvent acetone at 2.10 ppm, reference J. Org. Chem, 1997, 62(21), 7513)

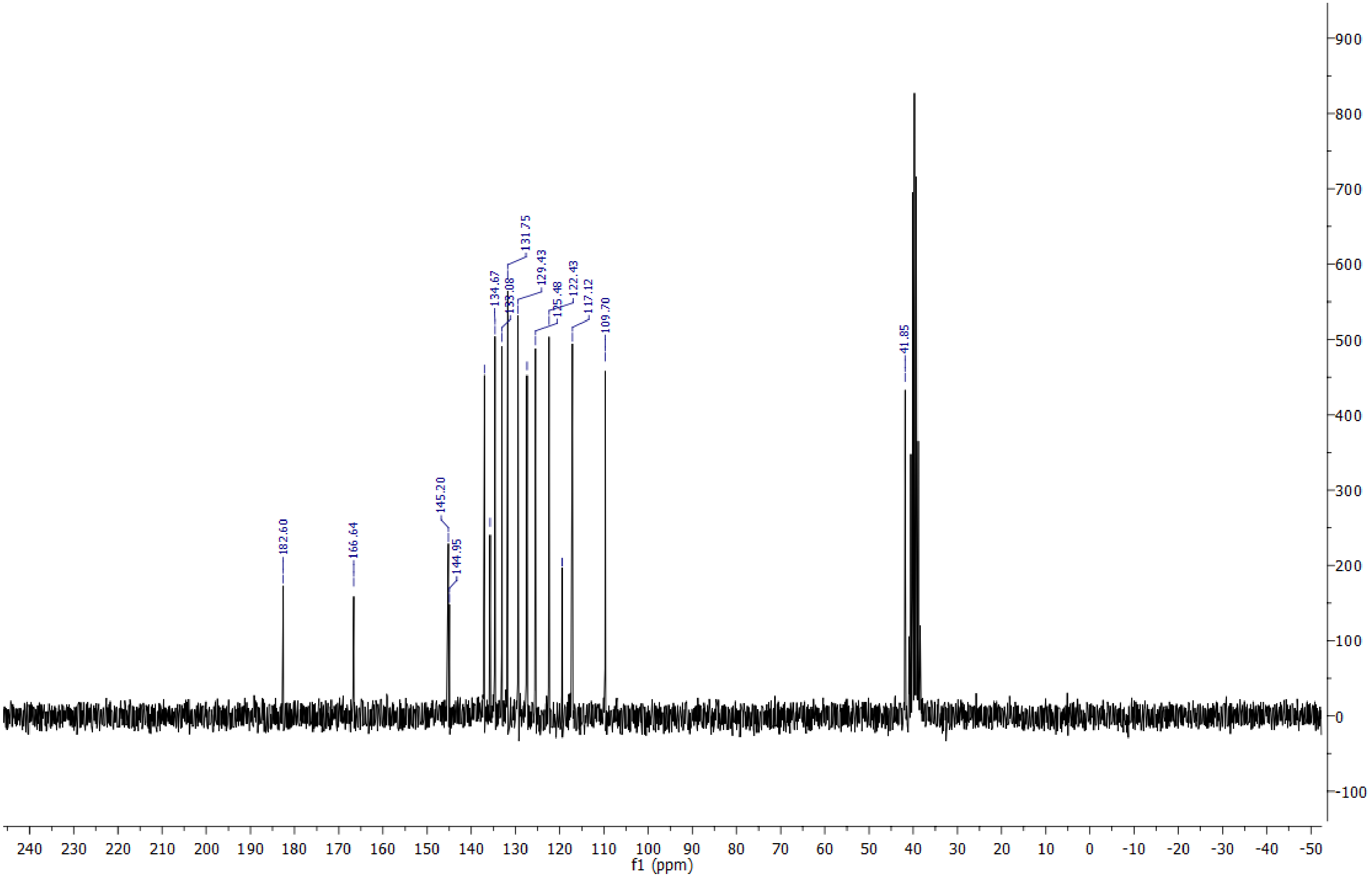

Figure S18. ^{13}C NMR spectrum of **4h**

3. MS spectra of 4a-h

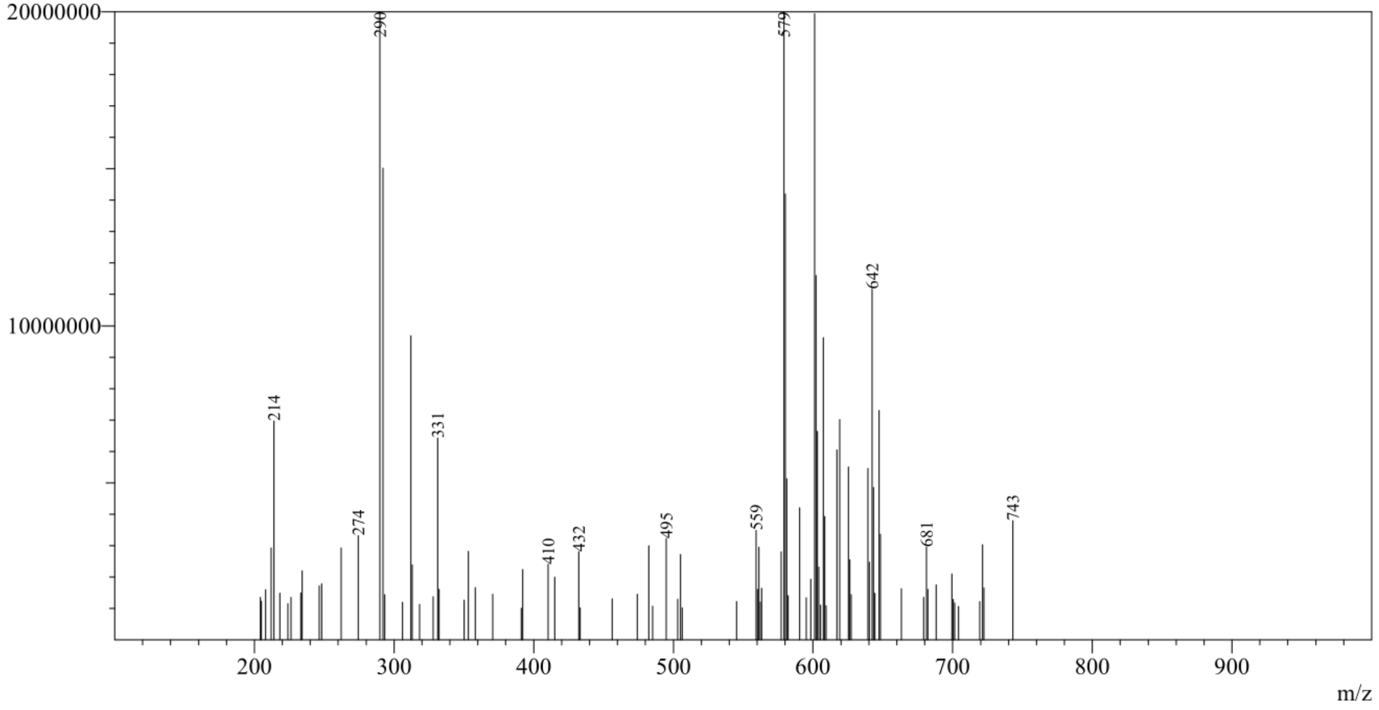

Figure S19. MS spectrum of **4a**

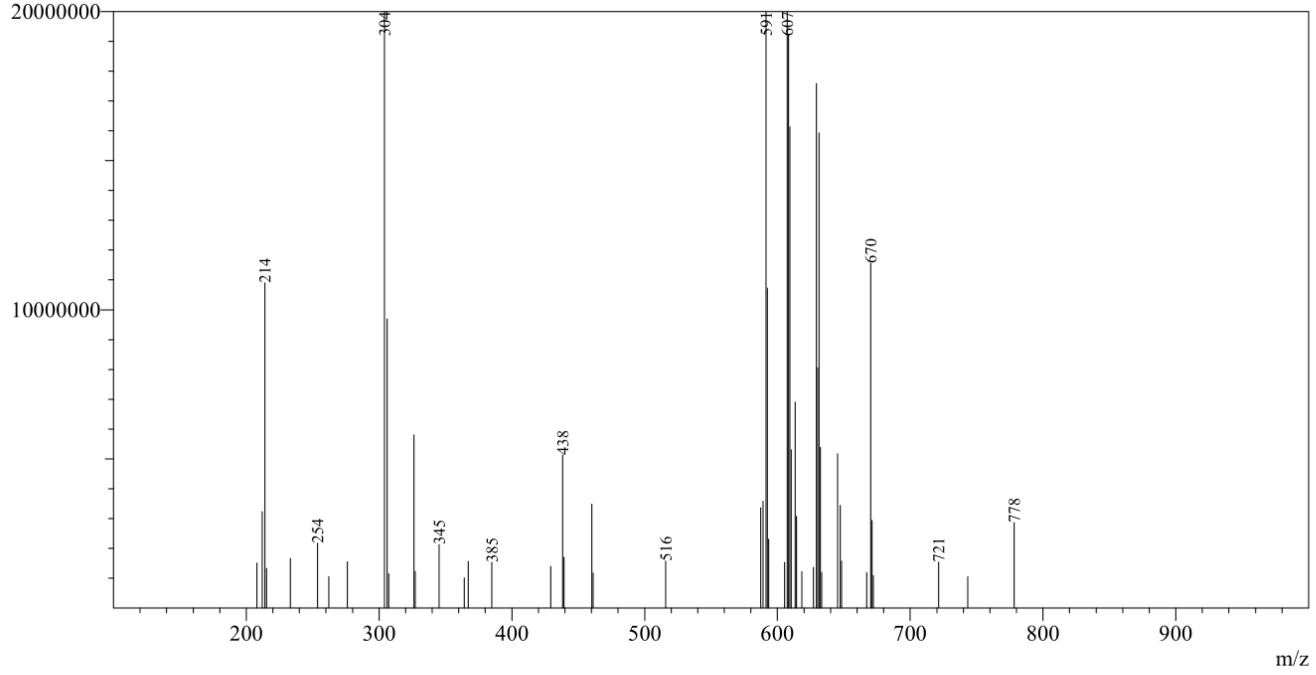

Figure S20. MS spectrum of **4b**

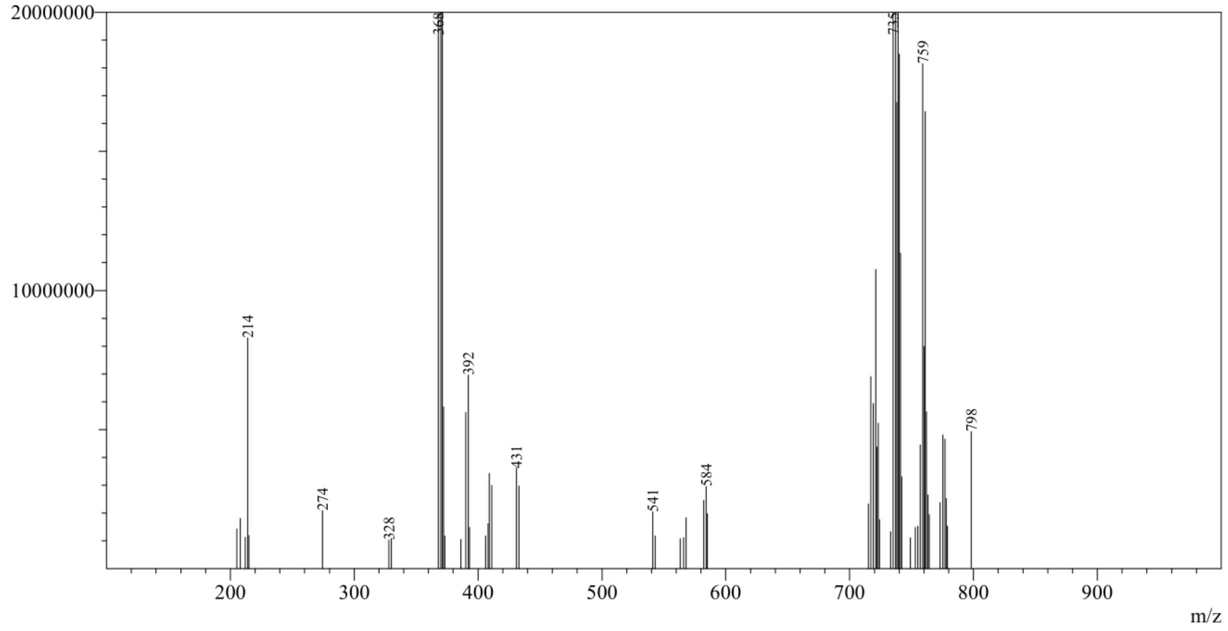

Figure S21. MS spectrum of **4c**

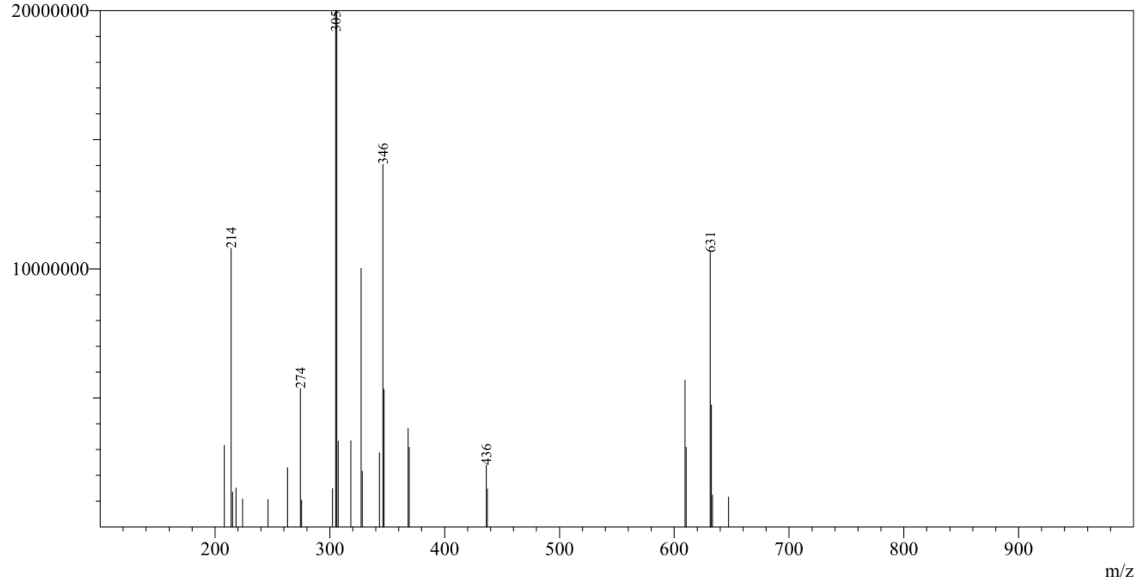

Figure S22. MS spectrum of **4d**

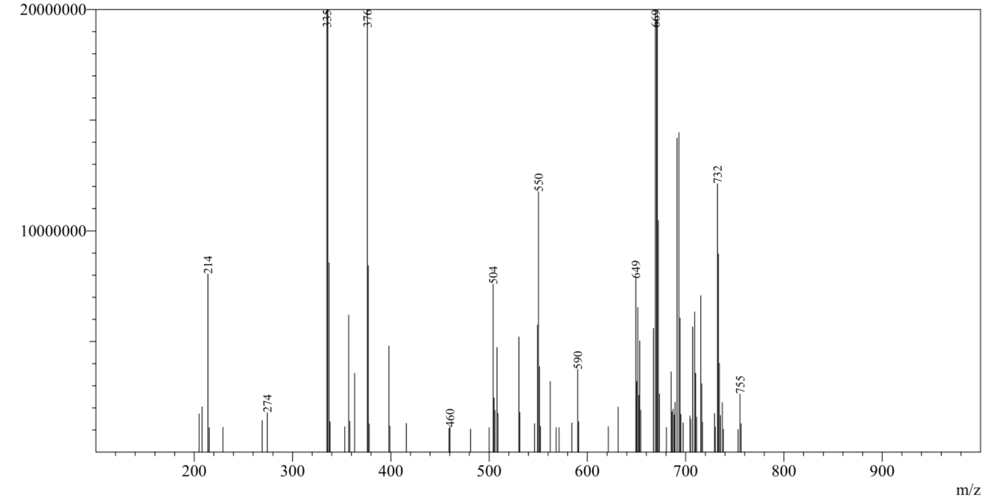

Figure S23. MS spectrum of **4e**

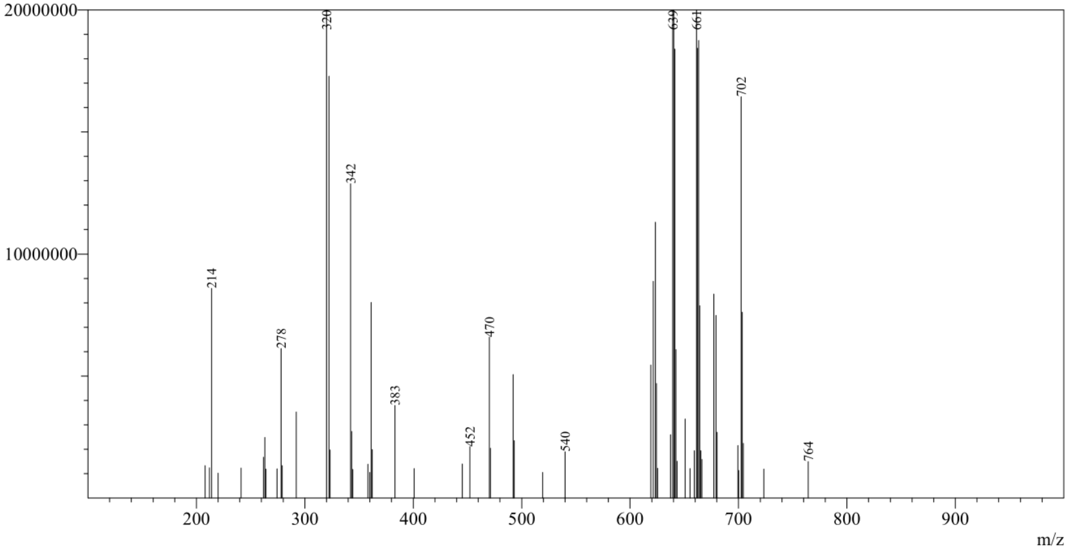

Figure S24. MS spectrum of **4f**

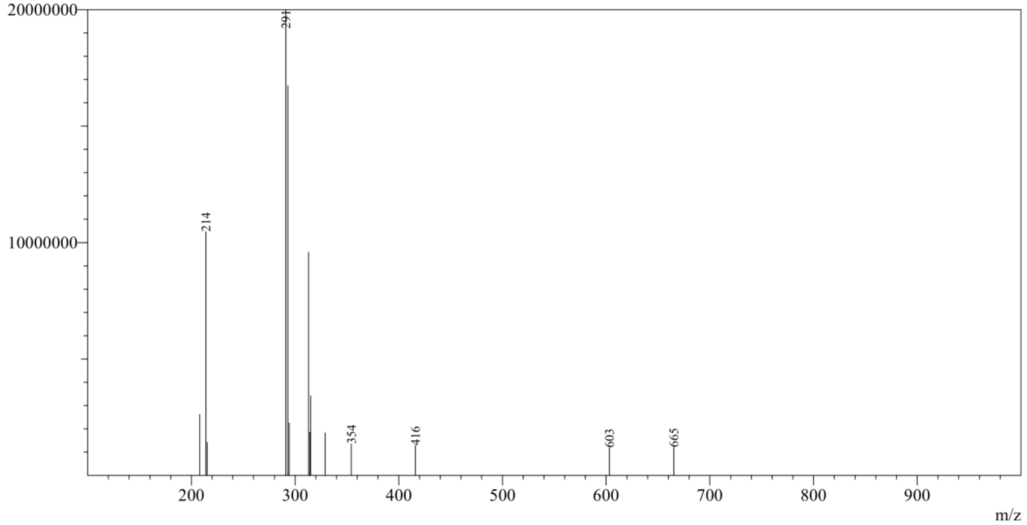

Figure S25. MS spectrum of **4g**

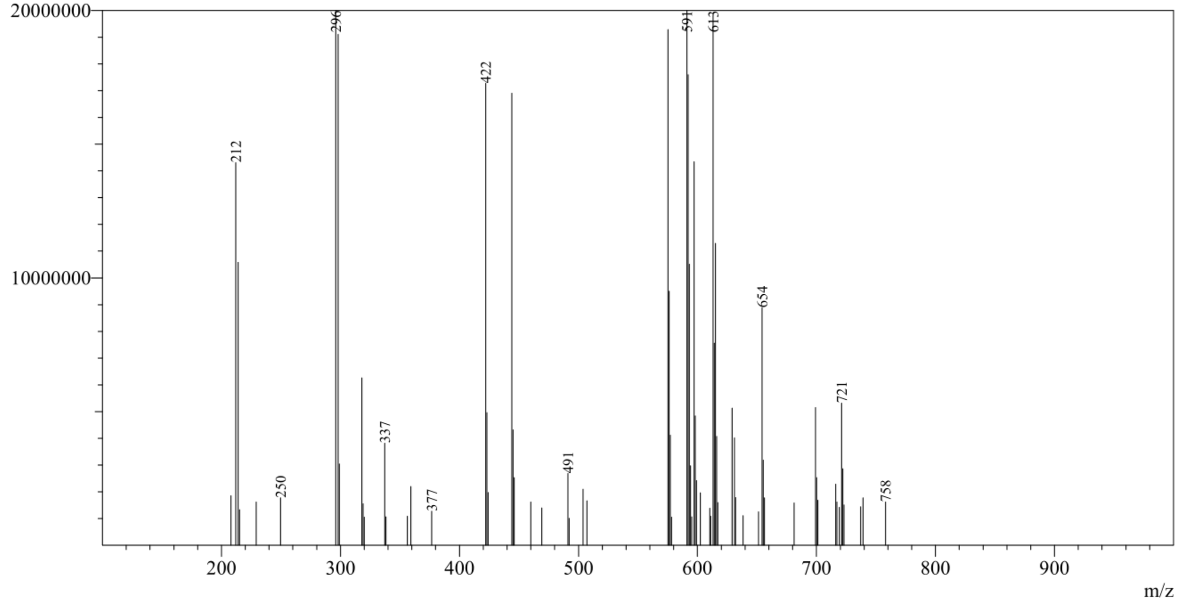

Figure S26. MS spectrum of **4h**

4. IR spectra of 4a-h

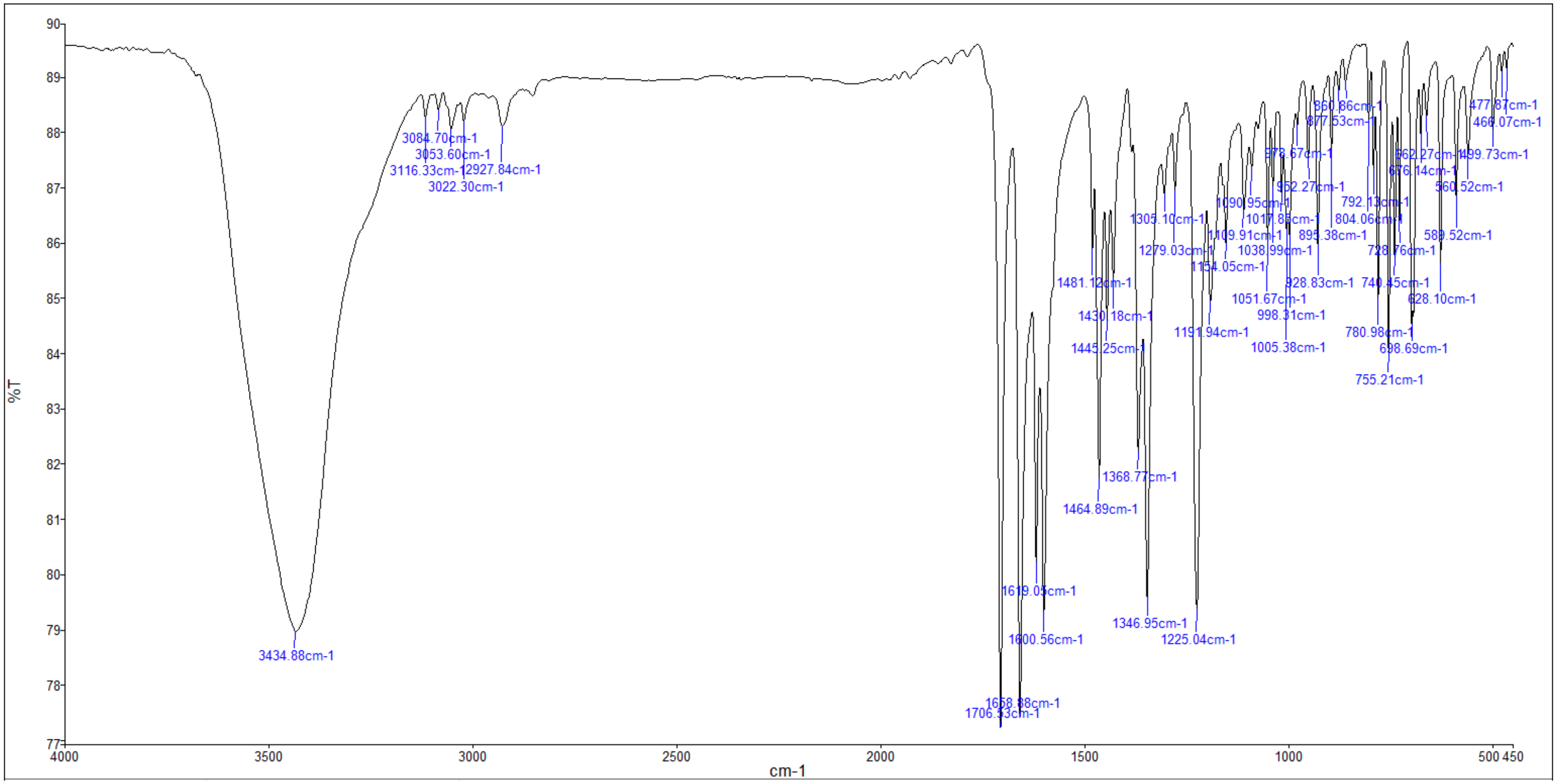

Figure S27. IR spectrum of **4a**

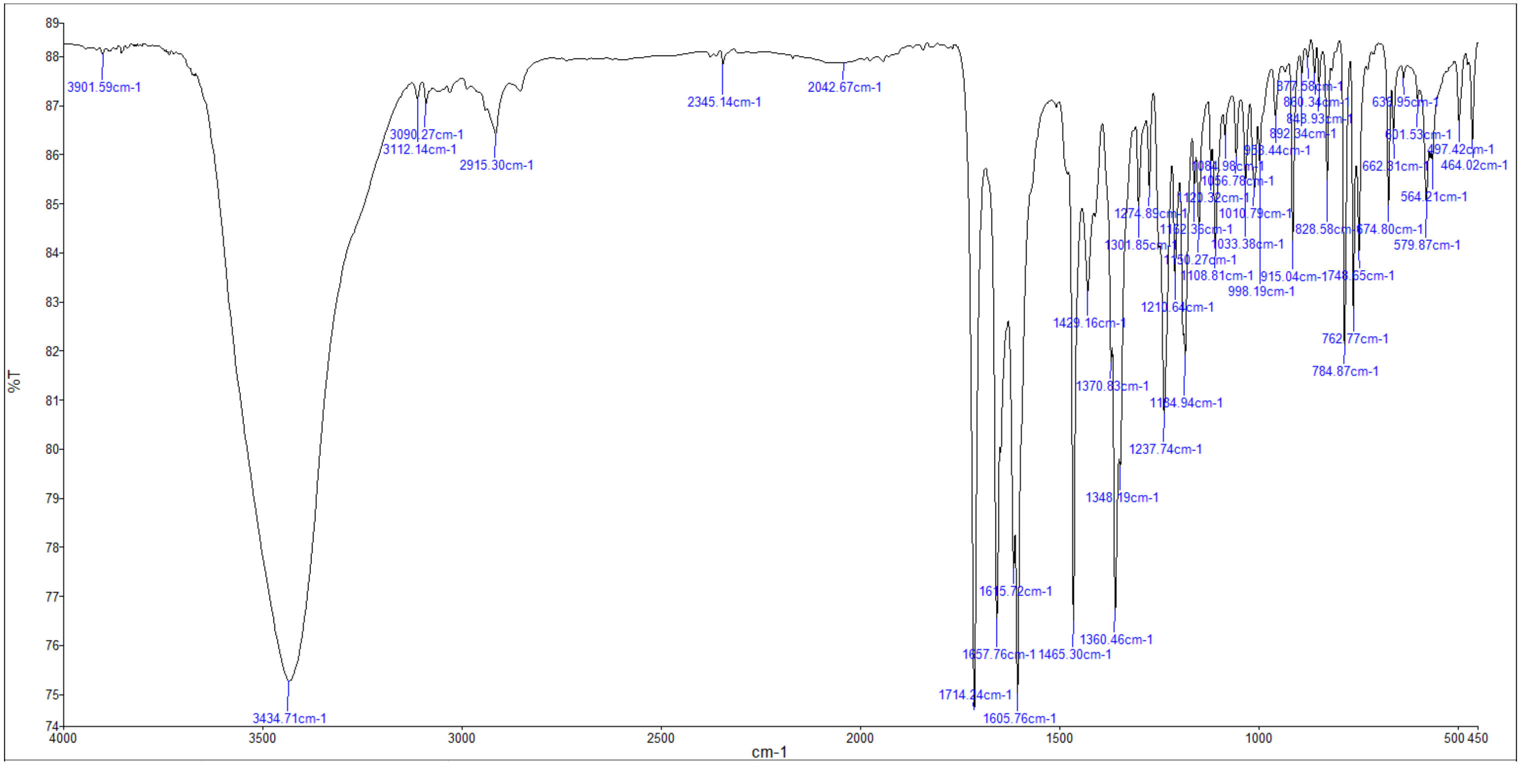

Figure S28. IR spectrum of **4b**

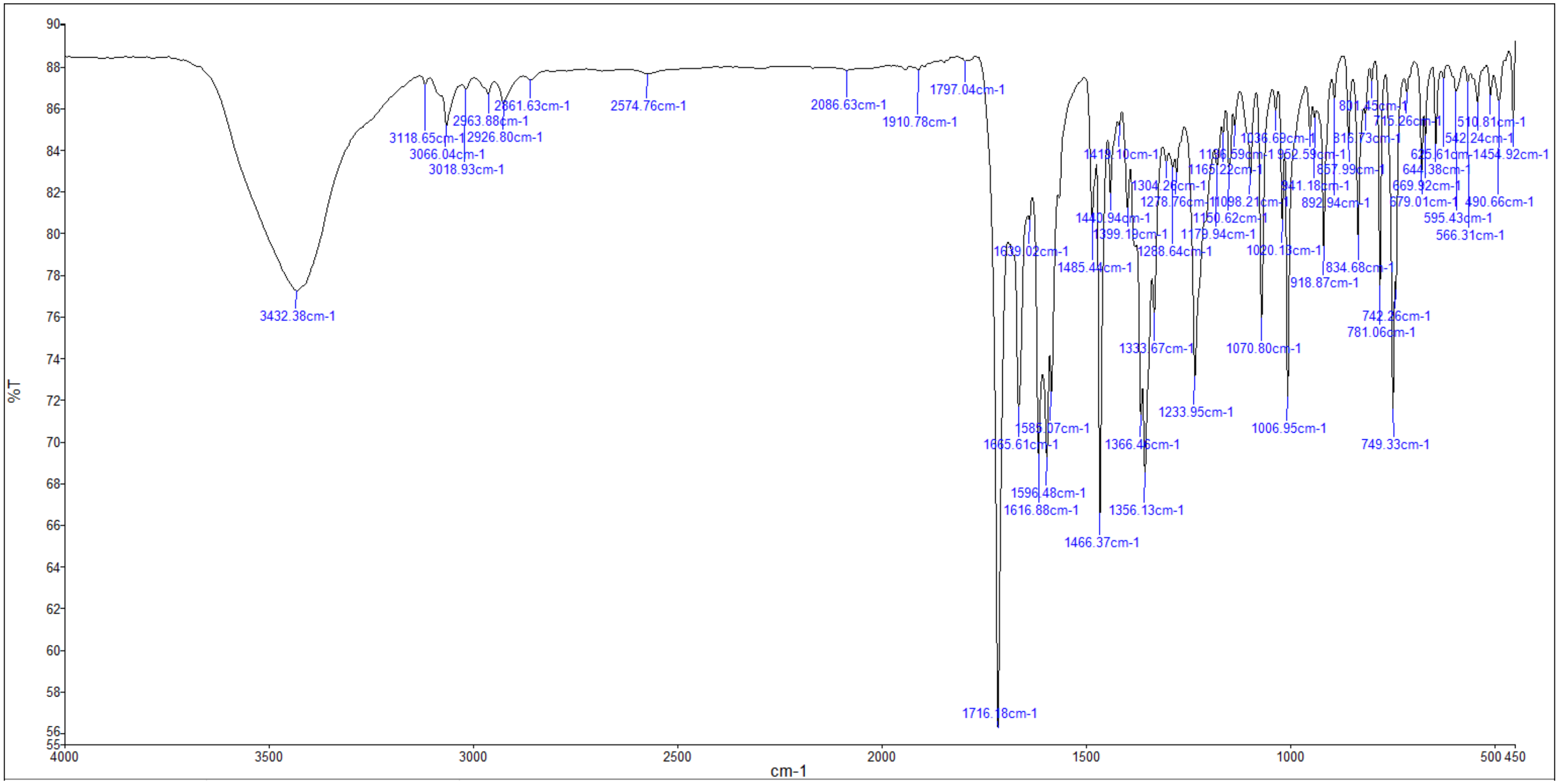

Figure S29. IR spectrum of **4c**

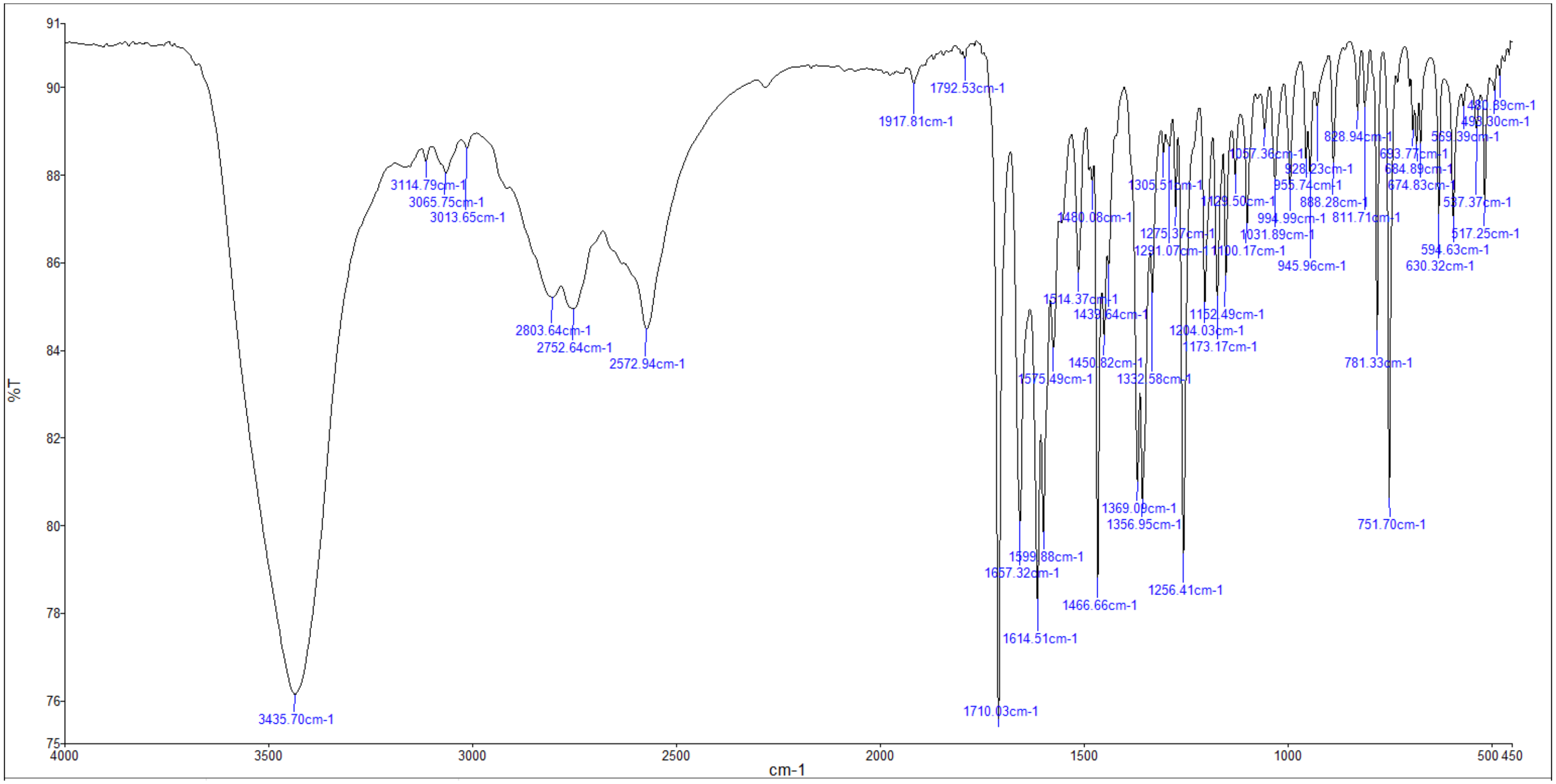

Figure S30. IR spectrum of **4d**

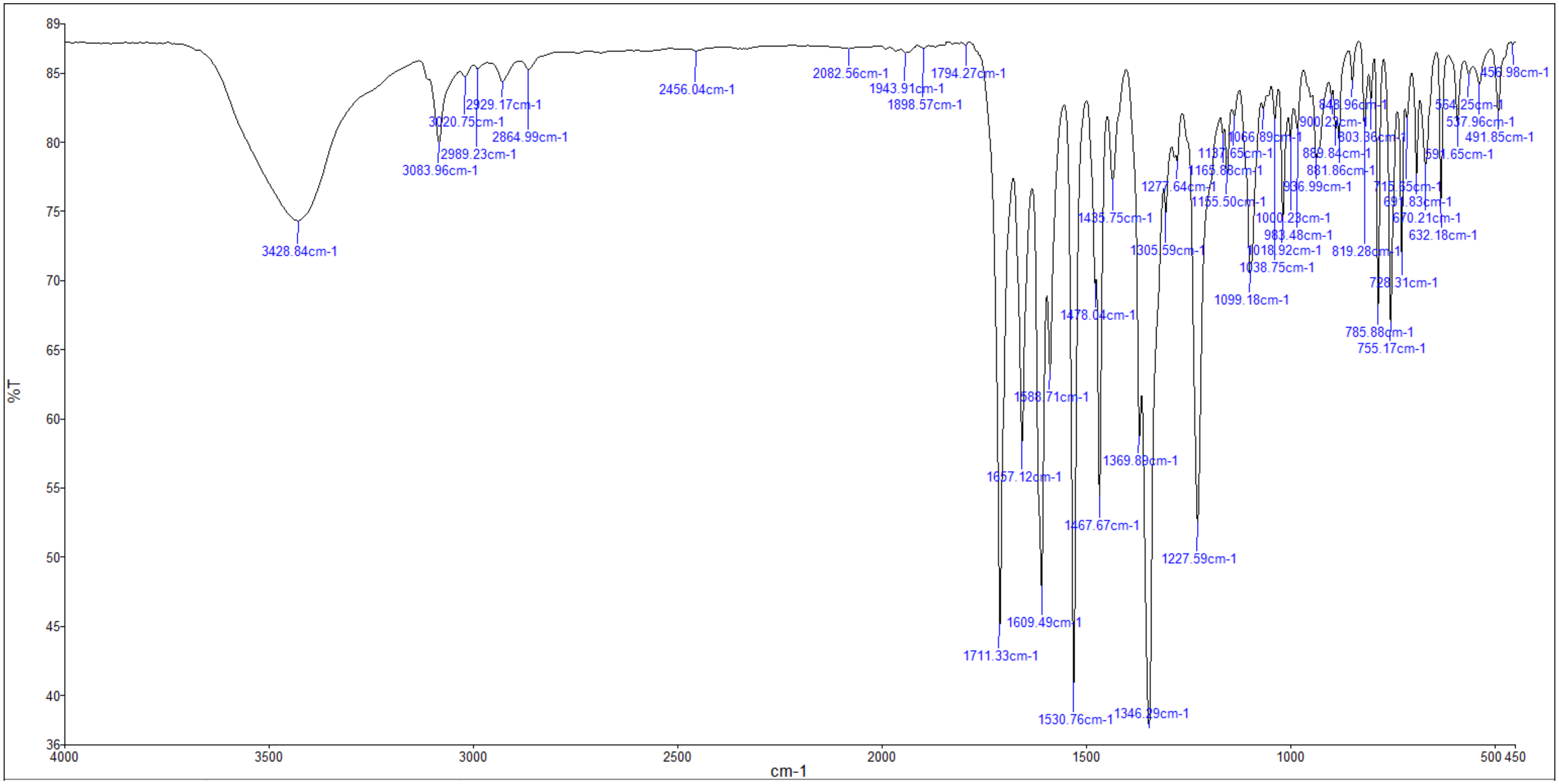

Figure S31. IR spectrum of **4e**

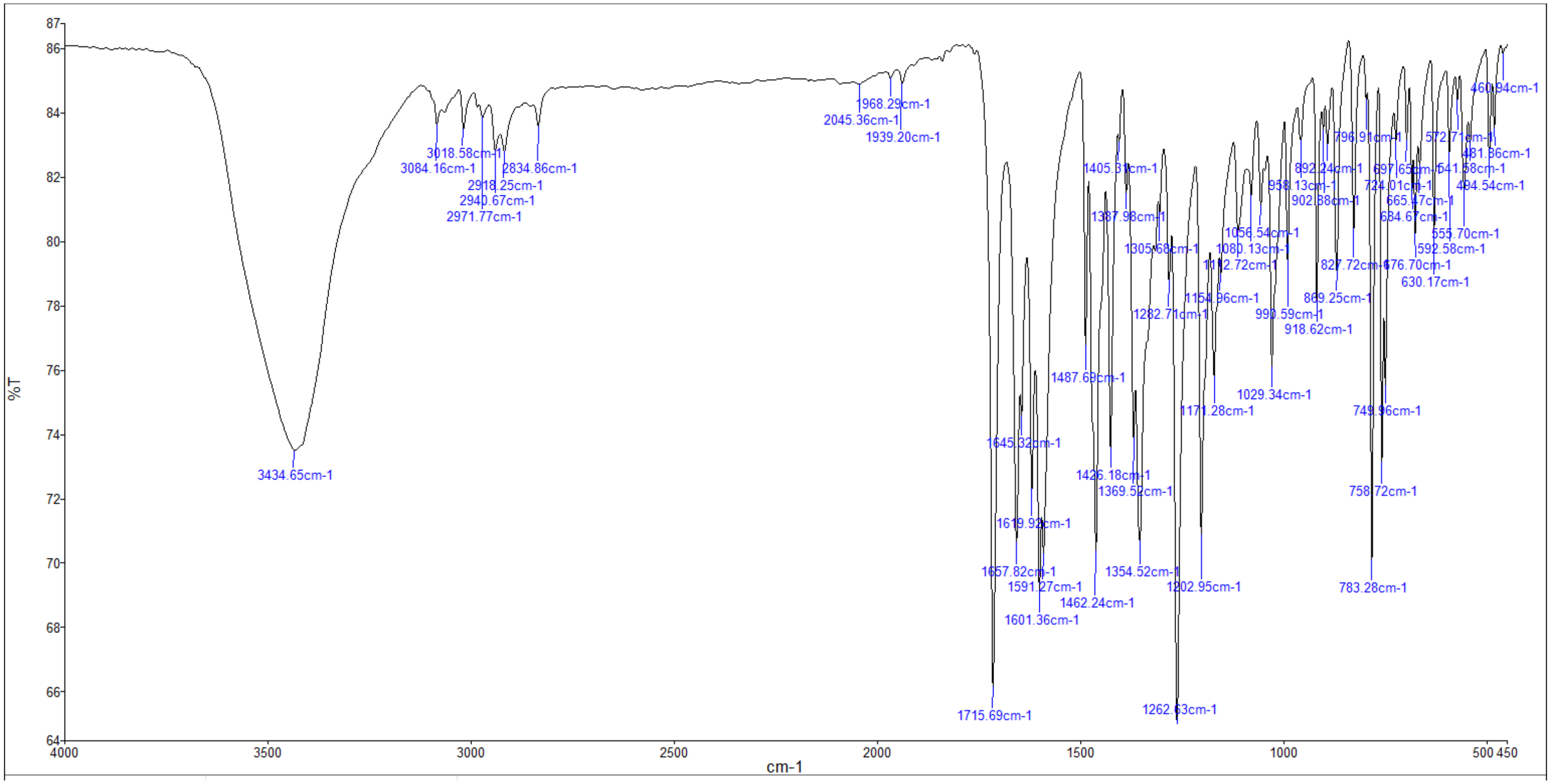

Figure S32. IR spectrum of **4f**

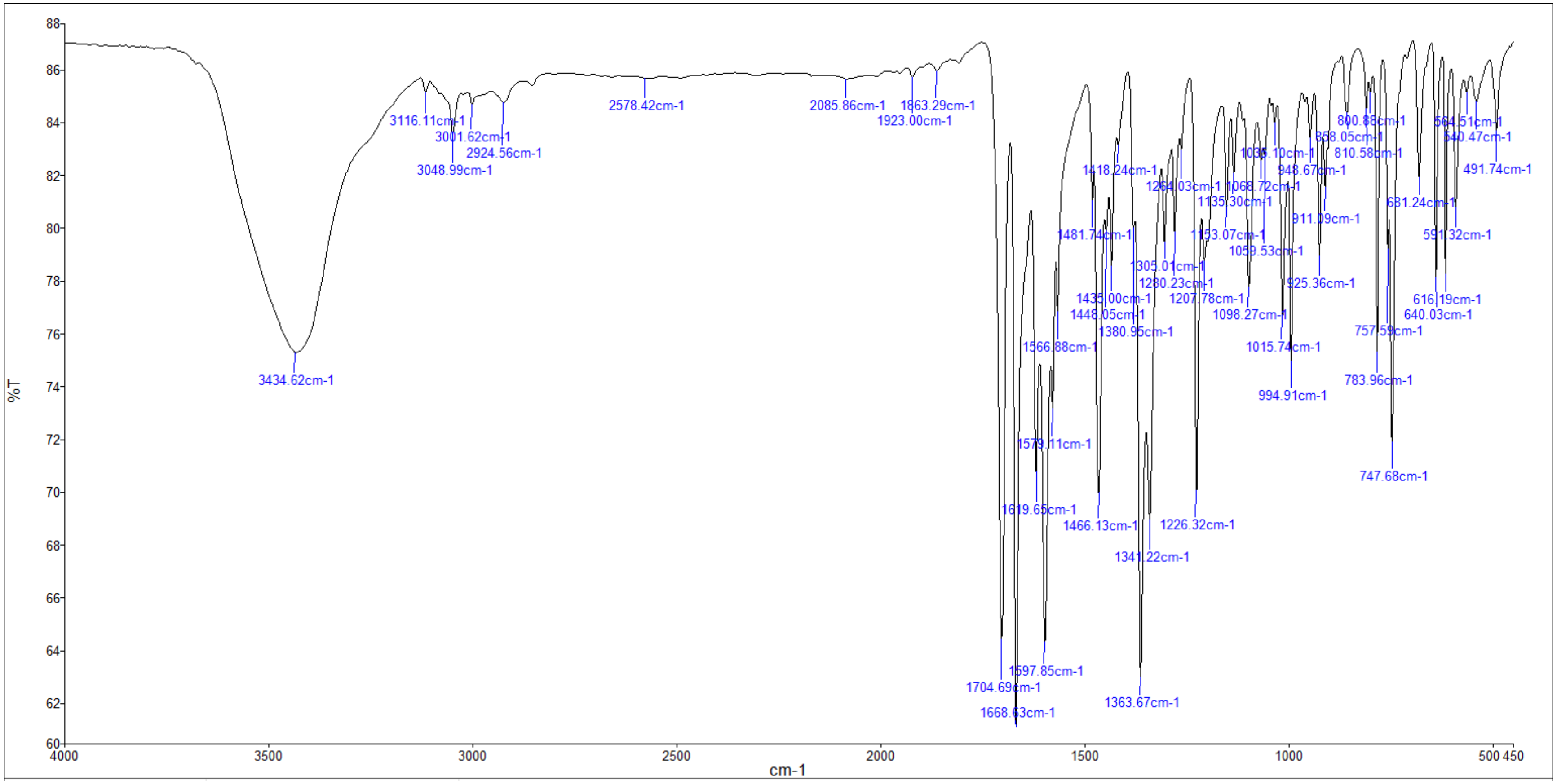

Figure S33. IR spectrum of **4g**

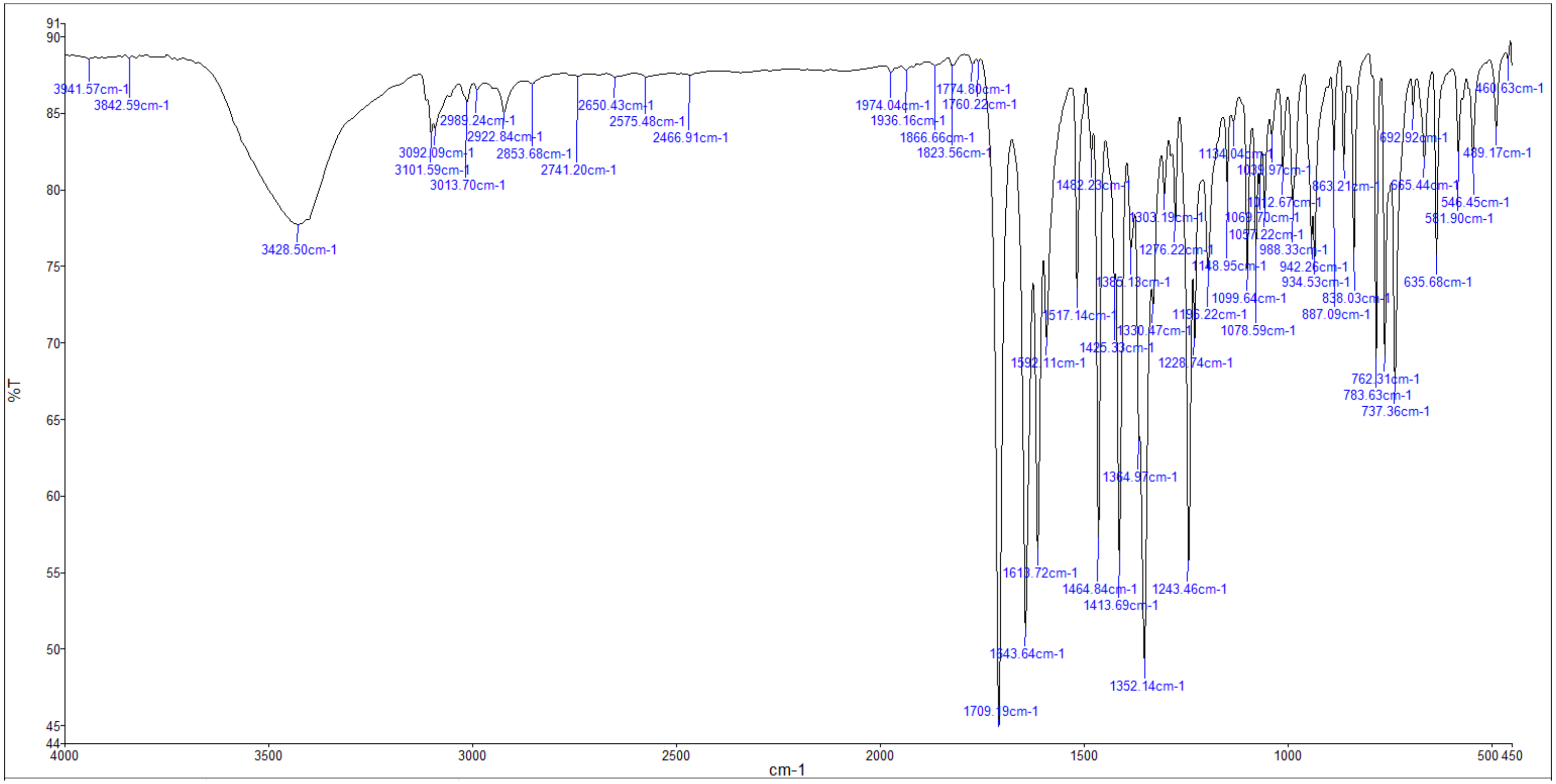

Figure S34. IR spectrum of **4h**
